# Validation and calibration of a novel GEM biosensor for specific detection of Cd^2+^, Zn^2+^, and Pb^2+^

**DOI:** 10.1186/s12896-023-00820-7

**Published:** 2023-12-08

**Authors:** H. M. L. P. B. Herath, W. R. M. de Silva, R. S. Dassanayake, Y. I. N. S. Gunawardene, J. R. P. Jayasingha, M. K. Gayashan, L. O. B. Afonso, K. M. N. de Silva

**Affiliations:** 1 Centre for Advanced Materials and Devices (CAMD), Department of Chemistry, Faculty of Science, University of Colombo, Colombo, 00300 Sri Lanka; 2 School of Life and Environmental Sciences, Faculty of Science, Engineering and Built Environment, Deakin University, Geelong, Australia; 3 Molecular Medicine Unit, Faculty of Medicine, University of Kelaniya, Ragama, Sri Lanka

**Keywords:** Microbial biosensors, Cadmium, Lead, Zinc, Genetic circuits, Heavy metal detection

## Abstract

**Background:**

In this study, we designed a novel genetic circuit sensitive to Cd^2+^, Zn^2+^ and Pb^2+^ by mimicking the *CadA/CadR* operon system mediated heavy metal homeostasis mechanism of *Pseudomonas aeruginosa*. The regular DNA motifs on natural operon were reconfigured and coupled with the enhanced Green Fluorescent Protein (eGFP) reporter to develop a novel basic NOT type logic gate *CadA/CadR-*eGFP to respond metal ions mentioned above. A Genetically Engineered Microbial (GEM)-based biosensor (*E.coli*-BL21:pJET1.2-*CadA/CadR-*eGFP) was developed by cloning the chemically synthesised *CadA/CadR-*eGFP gene circuit into pJET1.2-plasmid and transforming into *Escherichia coli* (*E. coli*)-BL21 bacterial cells.

**Results:**

The GEM-based biosensor cells indicated the reporter gene expression in the presence of Cd^2+^, Zn^2+^ and Pb^2+^ either singly or in combination. Further, the same biosensor cells calibrated for fluorescent intensity against heavy metal concentration generated linear graphs for Cd^2+^, Zn^2+^ and Pb^2+^ with the R^2^ values of 0.9809, 0.9761 and 0.9758, respectively as compared to non-specific metals, Fe^3+^ (0.0373), AsO_4_^3−^ (0.3825) and Ni^2+^ (0.8498) making our biosensor suitable for the detection of low concentration of the former metal ions in the range of 1–6 ppb. Furthermore, the GEM based biosensor cells were growing naturally within the concentration range of heavy metals, at 37 °C and optimum pH = 7.0 in the medium, resembling the characteristics of wildtype *E.coli*.

**Conclusion:**

Finally, the novel GEM based biosensor cells developed in this study can be applied for detection of targeted heavy metals in low concentration ranges (1–6 ppb) at normal bacterial physiological conditions.

**Supplementary Information:**

The online version contains supplementary material available at 10.1186/s12896-023-00820-7.

## Introduction

Heavy metals occur naturally in the environment and are generally characterized as metallic chemical elements with relatively high density that can exhibit toxicity or harmful effects at low concentrations [1]. Their extensive usage in various industrial, medical, agricultural, and household applications has resulted in their widespread distribution in the environment [2]. When released into water reservoirs, heavy metals tend to accumulate along food chains, leading to the eventual occurrence of heavy metal poisoning in higher vertebrates [3, 4]. Systemic toxins such as Arsenic (As), Lead (Pb), and Mercury (Hg) can adversely affect multiple organs or different cell types, even in small quantities [4]. The International Agency for Research on Cancer (IARC) classifies Cadmium (Cd), Chromium (Cr), Nickel (Ni), and Arsenic (As) as belonging to the group of 'Group 1 carcinogens,' denoting their potent ability to cause cancer [5].

The assessment of environmental risk resulting from heavy metal pollution has traditionally relied on quantifying total metal content through conventional analytical methods that involve the digestion of samples with strong acids [6]. Various chemical techniques are commonly employed for heavy metal detection, including chemical precipitation, ion exchange, chelation, membrane separation, cold vapor atomic absorption spectrometry, inductively coupled plasma mass spectrometry, UV–visible spectrometry, and X-ray absorption spectroscopy [7–9]. However, these techniques have significant limitations in terms of their applicability in field settings due to their complexity and the substantial time required to conduct the analysis. Consequently, there is currently a growing interest in the development of rapid and portable techniques for the detection of heavy metals in contaminated water samples [10].

Biosensors are analytical devices that combine bio components with transducers to convert biological signals into electrical signals, making them well-suited for real-time environmental monitoring [11]. Heavy metal detecting biosensors can be broadly categorized into two groups based on the nature of the biological components utilized: protein-based (including enzymes, metal-binding proteins, and antibodies) and whole-cell-based biosensors, which encompass both natural and genetically engineered microorganisms [11]. Biosensors offer several advantages for detecting environmental contaminants, including their specificity, affordability, user-friendliness, portability, and ability to provide continuous real-time signals [11, 12]. A key attribute of these biosensors is their capacity to detect "bioavailable" levels of heavy metals, which are closely associated with environmental risks and toxicity [13]. Overall, biosensors present an ideal alternative for the detection of heavy metal contamination in environmental samples.

One of the major disadvantages of typical biosensors is their non-specific responses to various environmental pollutants [14]. In order to overcome this drawback, biosensors can be genetically improved, incorporating specific molecular mechanisms with constructed genetic circuits. The most promising approach to construct gene circuits to detect heavy metals is to architect the mechanism inside a live microorganism. The molecular mechanisms are often enhanced in microbial cells due to the optimal environment provided by the cells [15, 16]. This category of biosensors is widely known as Genetically Engineered Microorganism (GEM) based sensors and typically rely on the analysis of gene expression by creating transcriptional fusions between a promoter of interest and the reporter gene expression. This serves as a measurement for the availability of specific pollutants in complex environments [17]. There have been numerous attempts to design GEM sensors for heavy metal detection by encoding protein components that are naturally occurring in bacteria to resist from heavy metals adapting metal sequestration and efflux mechanisms [18, 19].

The rapid development of genetically engineered biosensors has revolutionized the field of heavy metal detection and bioremediation. These biosensors offer precise and efficient means to monitor the bioavailability of heavy metals and enable the development of strategies for their remediation. In recent years, significant progress has been made in the utilization of bacterial cell-based biosensors for the detection and remediation of cadmium (Cd) contamination [20]. The genetics and phenotypic properties of extremophiles have provided valuable insights for the development of novel biological strategies for bioremediation, thereby contributing to the progress of grey biotechnology [21]. Moreover, whole-cell-based microbial biosensors (WCBMB) has been recognised as a crucial tool that integrates genetically engineered organisms with operator/promoter sequences derived from heavy metal-resistant operons, coupled with regulatory proteins in the gene circuit. The use of WCBMB has enabled effective monitoring of xenobiotics in situ, allowing for real-time detection and evaluation of environmental pollutants [22].

The utilization of GEMs as biosensors for heavy metal detection holds immense potential. However, the existing biosensors described in the literature, which are still in the experimental stages, lack crucial information regarding the physiology of the microorganisms used in these biosensing applications. Considering this, our study aims to develop an ideal and innovative GEM biosensor that is sensitive to specific heavy metals, with the future goal of testing it for on-site detection.

To accomplish this, we present the validation and calibration of a quantitative multi bacterial-cells digital tool designed to specifically detect Cd^2+^, Zn^2+^, and Pb^2+^ using bacterial cells. In our study, we have designed a novel genetic circuit by rearranging the natural *CadA/CadR* gene operon of *Pseudomonas aeruginosa*. This genetic circuit enables the selective sensing of Cd^2+^, Pb^2+^, and Zn^2+^ metal(loids). Additionally, we incorporated the enhanced Green Fluorescent Protein (eGFP) gene coding sequence as the reporter gene during the genetic circuit design stage.

To develop the genetically modified heavy metal-sensitive bacterial biosensor cells, the DNA construct-containing plasmid was chemically transformed into *Escherichia coli* (*E. coli*)—BL21 bacterial strain. The presence of the newly introduced genetic circuit in bacterial cells was initially confirmed using molecular techniques such as Polymerase Chain Reaction (PCR). Subsequently, the modified bacterial cells underwent growth and physiology validation tests. These tests involved culturing the modified cells in the presence of control heavy metals within the desired sensing range to evaluate their ability to reconstruct the typical sigmoid growth curve of bacteria without altering their normal physiology.

Further experiments confirmed the expression of the *eGFP* reporter gene in bacterial cells upon exposure to the heavy metals of interest using quantitative PCR (qPCR). We also verified the production of eGFP proteins in bacterial cells through fluorometry, by examining the fluorescent protein range, and visualized the green fluorescence in biosensing bacterial cells treated with corresponding heavy metals using fluorescence microscopy and the appropriate filters.

Before utilizing the genetically engineered bacterial cells in heavy metal fluorescent assessment assays, we examined their ability to provide optimal fluorescent signals under the *E. coli* bacteria's growth conditions, including pH, temperature, and specific incubation period.

Finally, we tested the engineered microbial strain's sensitivity and specificity towards Cd^2+^, Zn^2+^, and Pb^2+^ through digital image processing, using individual metal ion treatments as well as combinational metal treatments. We strongly believe that the genetically engineered heavy metal-sensitive bacterial biosensor cells will be potential candidates for future on-site heavy metal detection applications when coupled with digital devices. The validation and calibration approaches discussed in this study will also prove advantageous for future research in optimizing the integrity of GEM sensors.

## Material and methods

### Preparation of heavy metal solutions

The 100 ppm stock solutions of Cd^2+^, Pb^2+^, Zn^2+^, Ni^2+^, Fe^3+^ and AsO_4_^3−^ ions were prepared by using CdCl_2_, Pb(NO_3_)_2_, Zn(CH_3_COO)_2_, Ni(NO_3_)_2_.6H_2_O, FeCl_3_·6H_2_O and Na_2_HAsO_4_ (Sigma-Aldrich) respectively and dissolving them in ddH_2_O. The concentrations of each stock solution were confirmed initially by measuring the concentrations with Microwave Plasma-Atomic Emission Spectrometry (MP-AES) (Agilent 4210) [23]. Heavy metal standards of 0.1 ppm, 0.5 ppm, 1.0 ppm, 2.0 ppm, 3.0 ppm, 4.0 ppm and 5.0 ppm prepared from each metal by following a serial dilution method [24] and stored at room temperature for subsequent treatments. All the storage, handling, treatments, discarding of heavy metal contaminated samples and environmental releasing regulation were carried out by strictly following the standard Material Safety Data Sheet (MSDS) protocols as described by Sigma-Aldrich.

### Computational design and chemical synthesis of gene circuit

An enhanced novel artificial genetic circuit to efficiently sense targeted heavy metals (Cd^2+^, Zn^2+^ and Pb^2+^) was constructed by integrating the key DNA motifs of *Pseudomonas aeruginosa’s CadA/ CadR* operon, which exists naturally for the detoxification of cadmium ions [25]. To computationally draft the DNA architecture of the gene circuit, the main DNA sequences were retrieved from *Pseudomonas* genome database (https://www.pseudomonas.com/). The DNA motifs on the genetic circuit were coordinated to theoretically function as a NOT molecular logic gate as described by Chia-Hua Chuang [26] and the T7 protein produced by *E.coli*-BL21 genome was interlined with the circuit to trigger the transcription initiation of the molecular mechanism. In brief, the *T7* promoter coupled to the coding sequence of *CadR* gene (CP000744.1/PSPA7_1449) (WP_003154109) was placed in clockwise direction. A Ribosomal Binding Site (RBS) was embedded in between the *CadR* and *T7* promoter sequence as the transcription initiation recognition signal. The coding sequence of eGFP (DQ399412.1) coupled with *CadA* promoter (CP000744.1/PSPA7_1448) was incorporated in anti-clockwise direction. Both *CadR* gene and *eGFP* gene expression were terminated by two *T7* terminator sequences. The DNA architecture of the genetic circuit was visualized by SnapGene viewer software (http://www.snapgene.com). Eventually, the genetic circuit was chemically synthesized at Genscript, USA (https://www.genscript.com/) and was cloned into pJET1.2-plasmid (https://www.snapgene.com/resources/plasmidfiles/?set=basic_cloning_vectors&plasmid=pJET1.2). The cloned plasmid was designated as pJET1.2-*CadA/CadR.*

### Microbial strains, Bio-chemical construction and culture conditions

*E.coli*-BL21(DE3) (CP001509) bacterial strain was used as the host microorganism to provide the optimal physiological conditions for biosensor activity and to encapsulate the pJET-1.2-*CadA/CadR*. The competent cells of *E.coli*-BL21 (DE3) were prepared by using the chemical competent cell preparation method [27]. The plasmid containing genetic circuit was transformed into *E.coli*-BL21(DE3) chemically competent bacterial cells using the heat shock method [28]. Bacterial cells used in this study were grown on Luria–Bertani (LB) broth (10 g/L bacto-tryptone, 5 g/L bacto-yeast extract, and 5 g/l NaCl) supplemented with antibiotic (ampicillin, 100 mg/L) at 37 °C under vigorous shaking [29]. The pH of the medium was adjusted to pH = 7.0. Cultured cells were stored at 4 °C for short-term use and -20 °C for long term use.

### Polymerase Chain Reaction (PCR) confirmation of GEM cells

In developing GEM biosensor cells, the successful transformation of pJET1.2-*CadA/CadR* plasmids into *E.coli*-BL21 cells were confirmed by carrying out a colony PCR reaction with transformed bacterial cells [30]. Briefly, an isolated *E.coli*-BL21 transformed colony was picked with a sterilized tool and transferred into a PCR tube. The PCR reaction was performed in a 50 μL volume of PCR mixture by adding 5 μL of 10 × Ex Taq Buffer (with Mg^2+^), 4 μL of 2.5 mM dNTPs, 40 pmol of each primer, 50 ng of cDNA, and 5 U of Ex Taq polymerase (TaKaRa). PCR conditions were set as follows: initial denaturation at 94 °C for 3 min, there after 35 cycles of amplification at 94 °C for 30 s, 59 °C for 30 s, and 72 °C for 1.5 min, with a final extension at 72 °C for 5 min. Subsequently, the resulting PCR product was resolved on an agarose gel. Primer sequences are; *CadA/CadR*–F = 5′-*ATGAAGATCGGTGAACTGGCGA*-3′, *CadA/CadR*–R = 5′-*GCCAACCCTCCTCCAAT*-3′ and amplicon size is 652 bp. The chemically synthesized *CadA/CadR* genetic circuit was used as the positive control. The PCR buffer was used as the negative control. The plates containing PCR confirmed GEM cells were designated as *E.coli*-BL21(DE3)-pJET1.2-*CadA/CadR* and were sub-cultured every two weeks and stored in 4 °C cold room. The glycerol stocks containing *E.coli*-BL21 (DE3) -pJET1.2-*CadA/CadR* cells were stored in -70 °C freezers.

### Assessment of the effect of heavy metals on bacterial cell growth

The typical sigmoid shaped bacterial growth curve was reconstructed upon the presence of heavy metals in the medium with a modified protocol [31] to evaluate the toxicity effect of heavy metals on bacterial cells. Initially, a single colony of bacterial cells was inoculated in freshly prepared 15 mL of LB broth and the inoculated culture was incubated overnight at 37 °C and the pH of the medium was adjusted to 7.0. This culture was used as the starting culture and added 1 mL for 50 mL fresh LB broths treated to achieve effective concentrations of 1 ppb, 2 ppb, 3 ppb, 4 ppb, 5 ppb, 6 ppb and 1 ppm Cd^2+^ in the medium as the heavy metal to be tested. Absorbance readings at 600 nm were measured at 0, 1, 2, 3, 4, 5, 6, 7 and 8 h time points. An untreated, inoculated LB broth was used as the control experiment. A line graph was plotted between OD_600 nm_ Vs. time (hours) for each treatment separately.

### Quantitative Polymerase Chain Reaction (qPCR) for *eGFP* mRNA expression analysis

To monitor the production of *eGFP* mRNA upon the activation of gene circuit, a qPCR was performed and were reported in accordance with the Minimum Information for Publication of Quantitative Real-Time PCR Experiments (MIQE) [32]. Primers specific to the *eGFP* coding sequence were designed by using PrimerQuest Tool (Integrated DNA Technologies, USA) based on gene sequence provided by National Center for Biotechnology Information (NCBI) [http://www.ncbi.nlm.nih.gov] for *eGFP* gene sequence. Primer specificity was confirmed using primer-BLAST (https://www.ncbi.nlm.nih.gov/tools/primer-blast/). Primer sequences are eGFP-F = 5′- *GCTACCCCGACCACATGAA*- 3′ and eGFP-R = 5′-*GACGTTGTGGCTGTTGTAGT*—3′. The freshly cultured *E.coli*-BL21 cells were sourced for treatments as described in section "Microbial strains, Bio-chemical construction and culture conditions". Subsequently, cells were treated with Cd^2+^, Zn^2+^, Pb^2+^ and Ni^2+^ as a series of concentrations from 1.0 ppb to 6.0 ppb. The heavy metal treated cells were incubated for 2 h at 37 °C and subjected to RNA extraction using QIAprep Spin Miniprep Kit by following manufacturer’s protocol. The cDNA was prepared using iScript™ cDNA Synthesis Kit by following manufacturer’s protocol and aliquoted to prepare the qPCR reaction mixture of HOT FIREPol EvaGreen qPCR Mix Plus (ROX), 10.0 μL and cDNA template, 5.0 μL and reaction conditions were set to initial denaturation, 95 °C for 15 min; denaturation, 95 °C for 15 s; annealing. 59 °C for 20 s; extension, 72 °C for 20 s; for 40 thermal cycles (Biorad CFX96 Touch Real-Time PCR Detection System). The housekeeping gene of 18S ribosomal RNA (18SrRNA) was used as the reference gene.

### Fluorimetry for eGFP protein analysis

To investigate the correlation between heavy metal concentrations and fluorescent intensities generated due to the production of eGFP proteins, fluorimetry was used. The *E.coli*-BL21-pJET1.2-*CadA/CadR* cells were treated with Cd^2+^, Zn^2+^, Pb^2+^ and Ni^2+^ solutions at the concentration points of 1, 2, 3, 4, 5 and 6 ppb. Subsequently, the heavy metal treated cells were incubated for 2 h before subjecting to fluorometric readings. The fluorometric readings were recorded using Hitachi Fluorescent Spectrophotometer FL-7000 with a 96-well microplate reader accessory. The instrument parameters were set to excitation wavelength at 488 nm and the emission wavelengths in the range of 450– 550 nm. Triplicate measurements were obtained for each sample and the mean values were taken. The obtained relative fluorescence intensity (RFU) values were plotted against the metal ion concentrations in a line graph [33].

### Fluorescent microscopy

To visually observe the green fluorescent signals of bacterial cells, fluorescent microscopy was applied. *E.coli*-BL21(DE3)-pJET1.2-*CadA/CadR* cells and wildtype *E.coli*-BL21(DE3) cells exposed to 2 ppb of Cd^2+^, 2 ppb of Zn^2+^, 2 ppb of Pb^2+^, 2 ppb of Ni^2+^ and multiple heavy metal combinations for 2 h along with heavy metals untreated *E.coli*-BL21(DE3)-pJET1.2-*CadA/CadR* cells and wildtype *E.coli*-BL21(DE3) were harvested by centrifugation at 3500 × g for 5 min. at 4 °C. Subsequently, the cells were washed with phosphate buffered saline solution (PBS) and immediately resuspended in PBS solution supplemented with 0.3% as described by Ravikumar [29]. Microscopic slides were prepared in order to obtain a single layer of isolated cells [34]. Cells were observed under fluorescent microscope (Olympus BX53; magnification; 600X, Excitation wavelength range = 460–490 nm; filter; FITC) and the fluorescence images were captured and exported as JPG images.

### Digital image processing based fluorescent signal validation and calibration

#### Digital image processing

The fluorescent intensities of microscopic images were analysed using image processing software, ImageJ2 bundled with 64-bit Java 1.8.0_112 [35]. Briefly, three isolated single cells were selected from each image, and cell boundaries were marked with the freedom selection tool and then retrieved the Area, Mean Gray Value, Minimum/Maximum Gray Values, and Integrated Density. The Gray values were measured simultaneously for the immediate background of each cell. The calculated mean intensity values were then converted into Corrected Total Cell Fluorescent (CTCF) values by applying the equation described elsewhere [36]. The average CTCF values of all biological replicates were tabulated corresponding to the calibration graphs.

#### Validation of digital image processing based fluorescent signals

##### Assessment of fluorescent signals under optimal pH levels

The capability of *E.coli*-BL21(DE3)-pJET1.2-*CadA/CadR* cells to fluoresce significant signals under optimum pH conditions were examined by plotting the graph between pH values (2 – 12 pH) vs CTCF values. A single colony of *E. coli*-BL21 harbouring pJET1.2-*CadA/CadR* plasmid was grown overnight at 37 °C and pH in the medium 7.0. The overnight culture was diluted tenfold in minimal (M9) medium supplemented with 100 μg/mL ampicillin and incubated with 2.0 ppb of Cd^2+^, Zn^2+^, Pb^2+^, and Ni^2+^ in the medium in an orbital shaker at 200 rpm with a pH gradient from 2 – 12 pH for 2 h optimal incubation time and at the constant pH value of 7.0. The cells were subjected to fluorescent microscopy as described in section "Fluorescent microscopy" and image processing was done as described in section "Digital image processing" to quantify the pH dependent biosensing signals. Triplicate measurements were obtained for each sample and the mean value was taken.

##### Assessment of fluorescent signals in optimal temperature conditions

The capacity of *E.coli*-BL21(DE3)-pJET1.2-*CadA/CadR* cells to fluoresce significant signals in optimum temperature conditions was examined by plotting the graph between temperature of the medium (0 – 60 °C) vs CTCF values. A single colony of *E. coli*-BL21 harbouring pJET1.2-*CadA/CadR* plasmid was grown overnight at 37 °C in pH of the medium 7.0. The overnight culture was diluted tenfold in minimal (M9) medium supplemented with 100 μg/mL ampicillin and incubated with 2.0 ppb concentrations of Cd^2+^, Pb^2+^, Zn^2+^ and Ni^2+^ in the medium in an orbital shaker at 200 rpm with a temperature gradient of 0, 25, 37, 45 and 60 °C with 2 h of optimal incubation time. The cells were subjected to fluorescent microscopy as described in section "Fluorescent microscopy" and image processing was done as described in section "Digital image processing" to quantify the temperature dependent biosensing signals. Triplicate measurements were obtained for each sample and the mean values were taken.

##### Time dependent induction of digital fluorescent signals

The effect of heavy metal incubation time on the intensity of fluorescent signals were monitored by conducting Cd^2+^, Zn^2+^ and Pb^2+^ treatments at different time points of 0, 1, 2, 3, 4, 5, 6 and 7 h with the concentration gradient of 1, 2, 3, 4, 5 and 6 ppb. The fluorescent intensity data converted into CTCF values from each time point were plotted on 3 dimensional plots with the Z-axis representing fluorescent intensities in CTCF, the Y-axis representing heavy metal concentration in ppb and the X- axis representing Time (hours). In generating the plots, the multiple experimental data was saved in the table format in three separate.csv files dedicated for Cd^2+^, Pb^2+^ and Zn^2+^. To visualize the data in 3-Dimentions (3D), Jupyter Notebook IDE v6.1.1 (https://jupyter.org/) was used. The code was implemented in Python v3.7.7 (https://www.python.org/downloads/release/python-377/). Initially,.csv files (Comma Separated Values) containing data were loaded into data frames (https://pandas.pydata.org/pandas-docs/stable/reference/frame.html) for visualization using Pandas library v1.1.0 (https://pandas.pydata.org/). Subsequently, using Plotly library v4.9.0 (https://plotly.com/), data in data frames was visualized in 3D Surface Plots.

### Calibration of concentration dependent heavy metal corresponding digital fluorescent signals

#### Individual heavy metal treatments

To observe the sensitivity of fluorescent signals of *E.coli*-BL21(DE3)-pJET1.2-*CadA/CadR* cells to single heavy metal treatments, the heavy metal concentration series were separately prepared for Cd^2+^, Zn^2+^, Pb^2+^, Ni^2+^, Fe^3+^ and AsO_4_^3−^ in the concentration range of 1 ppb, 2 ppb, 3 ppb, 4 ppb, 5 ppb and 6 ppb. The freshly cultured *E.coli*-BL21(DE3)-pJET1.2-*CadA/CadR* cells were sourced as described in the section "Microbial strains, Bio-chemical construction and culture conditions". Subsequently, the cells were incubated with individual heavy metal series for 2 h at 37 °C in the pH of 7.0 without shaking. Then, cells were subjected to fluorescent microscopy as described in section "Fluorescent microscopy" and images generated were exported and processed as described in the section "Digital image processing", respectively to measure biosensing signals. Triplicate measurements were obtained for each sample and the mean values were taken. Data were plotted on Corrected Total Cell Fluorescent (CTCF) Vs Heavy metal concentration [ppb] graphs to evaluate correlations of fluorescent signals for individual heavy metal treatments.

#### Heavy metal mixture treatments

The trends of heavy metal synergistic effects were observed by subjecting *E.coli*-BL21(DE3)-pJET1.2-*CadA/CadR* cells to Cd^2+^, Pb^2+^, Zn^2+^ combination treatments. The heavy metal mixtures were freshly prepared by combining corresponding heavy metals in 1:1 ratio, with the individual metal concentrations of 1 ppb, 2 ppb, 3 ppb, 4 ppb, 5 ppb and 6 ppb, to obtain the mixtures of [Cd^2+^  + Pb^2+^], [Pb^2+^ + Zn^2+^], [Cd^2+^  + Zn^2+^], and [Cd^2+^  + Zn^2+^  + Pb^2+^]. The freshly cultured *E.coli*-BL21(DE3)-pJET1.2-*CadA/CadR* cells were sourced as described in the section "Microbial strains, Bio-chemical construction and culture conditions". Subsequently, the cells were incubated with individual heavy metal series for 2 h at 37 °C in the pH of 7.0 without shaking. Then, cells were subjected to fluorescent microscopy as described in section "Fluorescent microscopy" and images generated were exported and processed as described in the section "Digital image processing", respectively to measure biosensing signals. Triplicate measurements were obtained for each sample and the mean values were taken. Data were plotted on Corrected Total Cell Fluorescent (CTCF) Vs Heavy metal concentration [ppb] graphs to evaluate correlations of fluorescent signals for heavy metal mixtures. Subsequently, the results obtained from the treatments involving heavy metal mixtures were further compared with those acquired from individual heavy metal treatments in a separate analysis.

### Statistical analysis

The GraphPad Prism 9 was employed for conducting the One-way analysis of variance (ANOVA) to analyse the data. The dependent variable in the analysis was the expression level, while the independent variables encompassed the heavy metal ion concentration and type. The least significant difference (LSD) post-hoc test was employed to assess the significant differences among the various metal ions, employing a 95% confidence interval (CI) to determine statistical significance. A significance level of *P* < 0.05 was set as the threshold for detecting significant differences.

## Results and discussion

### The genetic circuit and heavy metal sensing mechanism

Biosensors utilizing genetically engineered microorganisms have garnered significant attention over conventional analytical techniques due to their practical on-site applicability in environmental toxicity assessment [37–39]. By screening for upregulated or downregulated genes in the transcriptional profiles of heavy metal-treated bacterial cells, specific genes for selective heavy metal sensing can be identified [6, 40–42]. The *CadA/CadR* operon of *Pseudomonas aeruginosa* (*P. aeruginosa*) is well-known for its role in metal tolerance and homeostasis mechanisms in bacteria [43, 44]. A previous in-silico promoter analysis study demonstrated its specific response to Cd^2+^ [45], and Metal Responsive Elements (MREs) in the promoter overlapping regions have been shown to be specific for Cd^2+^, Pb^2+^, and Zn^2+^ [46]. A biosensor developed using the *Cad/CadC* promoter system in another bacterial strain (*Staphylococcus aureus*) showed sensitivity to detect Cd^2+^, Pb^2+^, and Sb^3+^ with low detection limits [6].

**Fig. 1:**
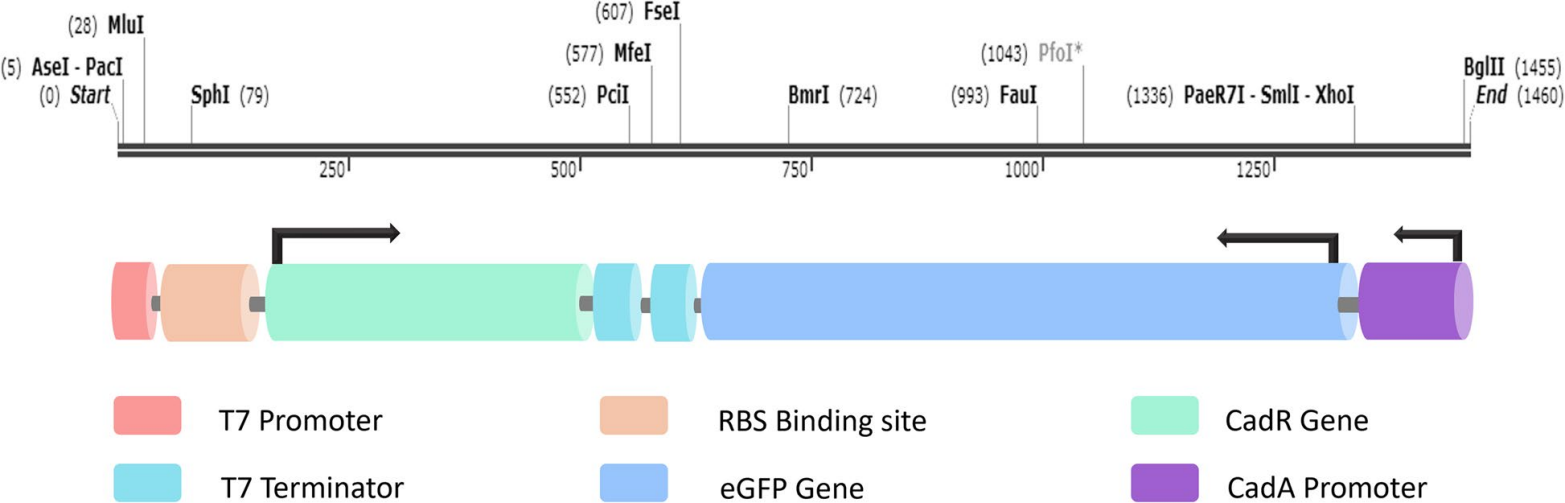
The genetic architecture of heavy metal sensitive *CadA/CadR* gene circuit. The *CadA* gene promoter is represented by a purple colour cylinder. The *T7* promoter is indicated by a dark pink colour cylinder. The *CadR* gene is depicted light green colour cylinder, while the *eGFP* gene is represented by blue colour cylinder. The Ribosomal Binding Site (RBS) is denoted by a light brown colour cylinder, and the *T7* terminators are shown as light blue colour cylinders. The DNA construct spans 1460 base pairs (bp) from the start site (0) to the end site (1460). Various restriction sites are incorporated, including AseI and PacI at the beginning, MluI at the end of the *T7* promoter, SphI at the end of the RBS, PciI at the end of *CadR*, MfeI at the end of the first *T7* terminator sequence, FseI at the end of the *eGFP* promoter, and PaeR7I, SmlI, and XhoI at the start of the *eGFP* gene coding sequence, as well as BglII at the start of the *CadA* promoter

**Fig. 2:**
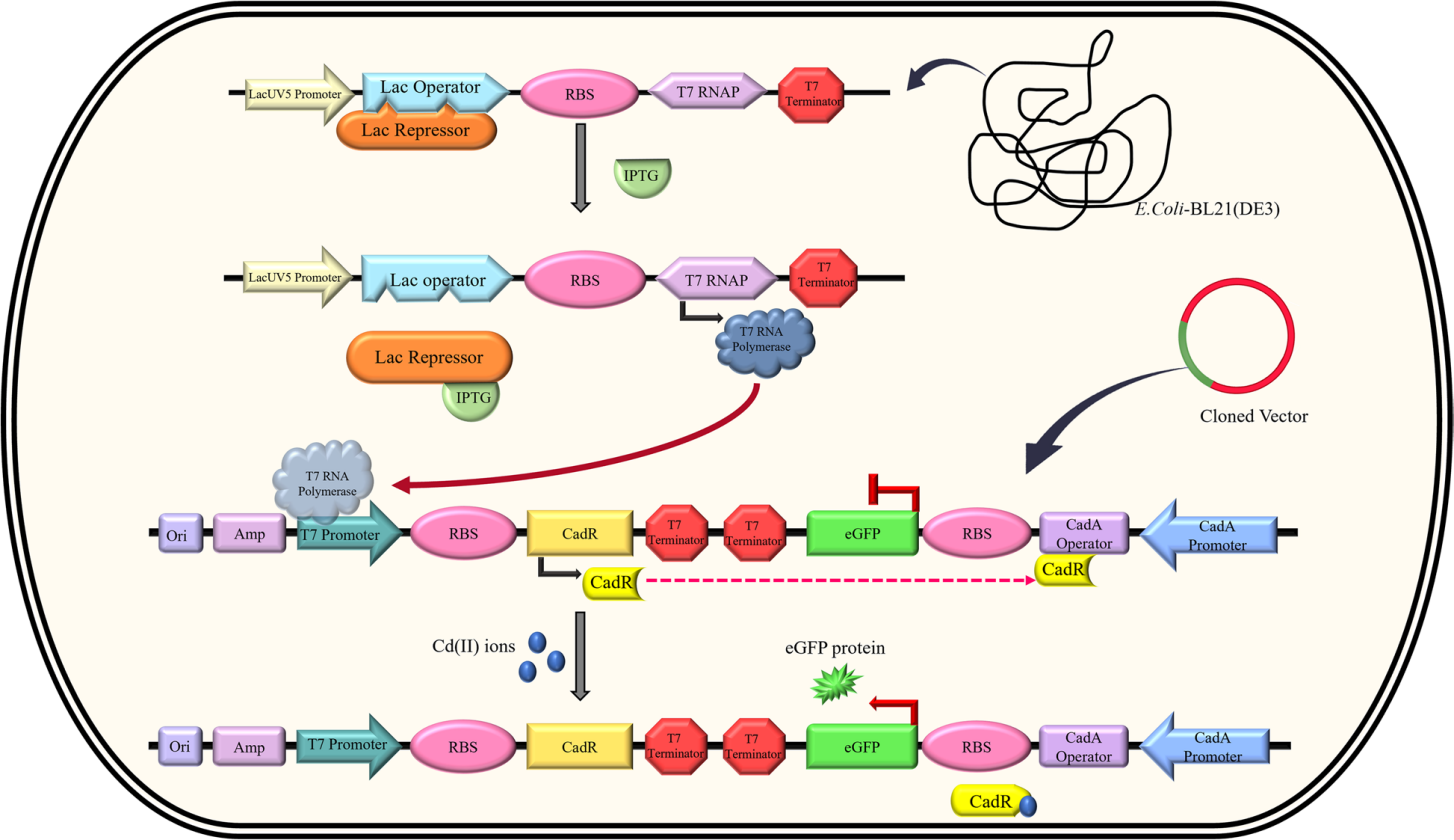
The heavy metal sensing mechanism of genetically engineered bacterial cells. The lacUV5 promoter controls the T7 RNA polymerase gene in the *E. coli*-BL21 genome. The Lac repressor binds to the Lac operator, repressing its activity. Upon the addition of IPTG inducer, the Lac repressor dissociates from the Lac operator. Subsequently, T7 polymerase is synthesized by the *E. coli* genome. This leads to the expression of the *CadR* gene by T7 polymerase, and the CadR protein binds to the *PcadA* operator, resulting in the repression of eGFP expression. In the presence of Cd^2+^ ions (green capsular symbol), the CadR protein undergoes a conformational change due to Cd^2+^ ion binding, causing its dissociation from the PcadA operator. This complete process allows for the expression of eGFP protein specifically in the presence of the corresponding metal ions, such as Cd^2+^ (illustrated by Cd.^2+^)

Based on this collective evidence, we selected the *CadA* and *CadR* genes of *P. aeruginosa* to construct a DNA sequence for Cd^2+^, Pb^2+^, and Zn^2+^ sensing in our current study. The genetic circuit employed key DNA motifs, including the *CadR* gene coding sequence of *P. aeruginosa*, the *CadA* gene promoter sequence of *P. aeruginosa*, the coding sequence of the eGFP protein, and the DNA sequence corresponding to the *T7* promoter. The total DNA sequence length was 1460 bp, comprising *CadR* (470 bp), *CadA* (114 bp), *eGFP* (720 bp), and *T7* (19 bp) (Fig. 1). The gene construct functioned as a NOT molecular logic gate to detect Cd^2+^, Pb^2+^, and Zn^2+^. NOT molecular logic gates act as ON–OFF switches, expressing the reporter gene in the presence of effector molecules and terminating expression when the effector molecules are absent [47]. The heavy metal sensing mechanism was distributed between the plasmid DNA and *E. coli*-BL21 genomic elements. The *T7* RNA polymerase, available under the control of the *lacUVS* promoter on the *E. coli*-BL21 genome, efficiently initiated gene transcription in the genetic circuit. The modified pJET1.2 plasmid vector contained key genetic components for metal sensing, with the *CadR* gene sequence regulated by the *T7* promoter, thereby controlling the expression of *CadR*. The *T7* promoter facilitated efficient transcription of the *CadR* gene, enabling its protein to interact specifically with Cd^*2*+^, Pb^2+^, and Zn^2+^ and induce conformational changes. The conformationally altered CadR proteins then targeted the *eGFP* gene segment, coupled with the *CadA* promoter, initiating the expression of fluorescent signals corresponding to the heavy metal doses (Fig. 2).

The pJET1.2-*CadA/CadR* plasmids in transformed bacterial cells were confirmed by the presence of a 652 bp long PCR product on agarose gel. No bands were detected in the wells corresponding to non-transformant *E.coli-*BL21(DE3) cells and negative control PCR products (Supp. Fig. 1). This confirms the efficient transformation of pJET1.2-*CadA/CadR* plasmids into *E.coli* -BL21(DE3) cells.

### The growth of *E.coli*-BL21(DE3)-pJET1.2-*CadA/CadR* cells

**Fig. 3:**
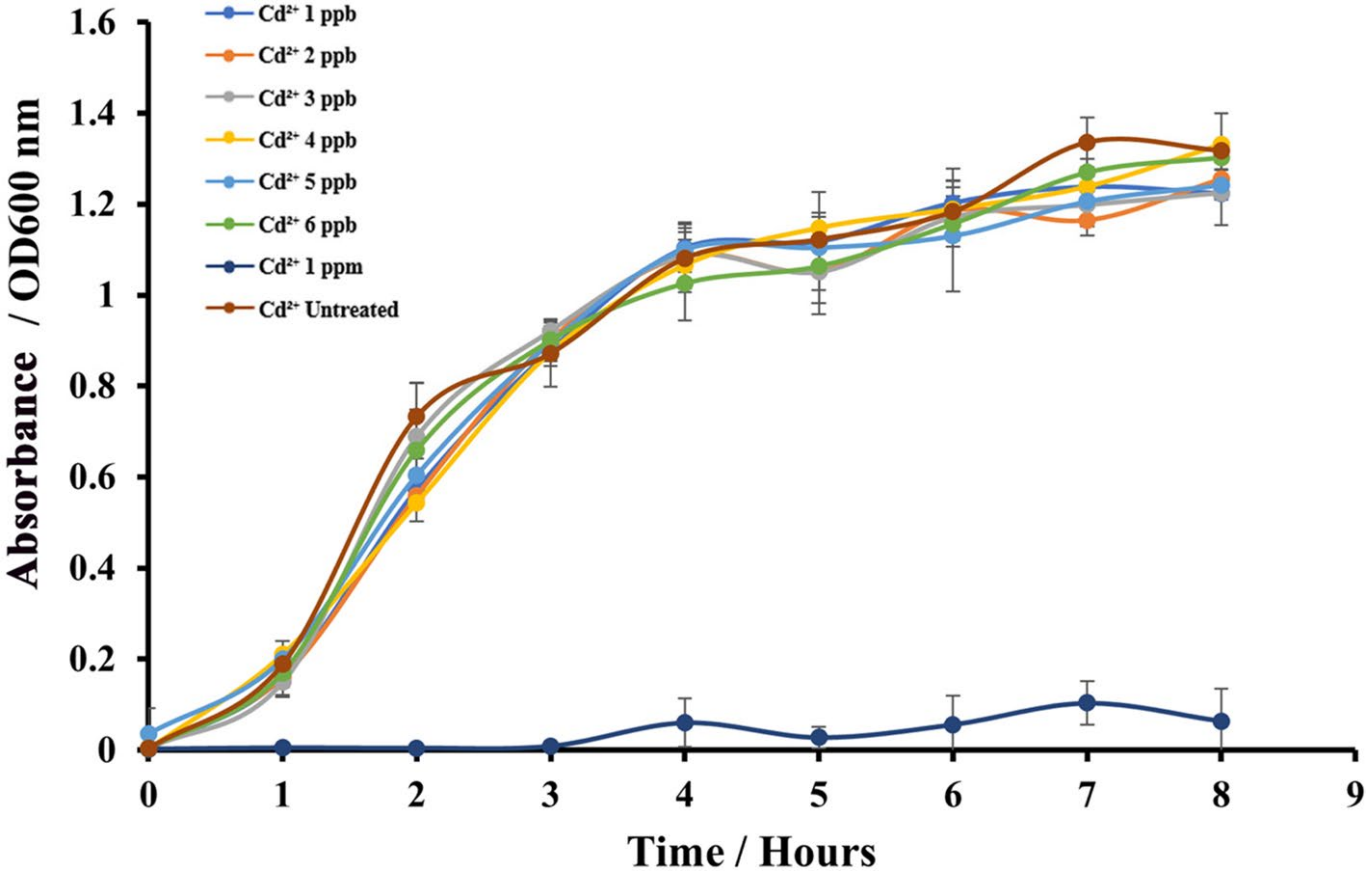
Absorbance at 600 nm over time for Cd^2+^-treated *E.coli*-BL21-pJET2.1-*CadA/CadR*-eGFP cells. Absorbance readings were measured at different time points (1–8 h) after treating cells with varying concentrations of Cd^2+^ ions (1 ppb, 2 ppb, 3 ppb, 4 ppb, 5 ppb, 6 ppb, 1 ppm and Cd^2+^-untreated) and compared to untreated cells. Each concentration is represented by a specific colour (blue, orange, ash, yellow, light blue, green, dark blue and brown) and the X-axis indicates time in hours. The Y-axis represents absorbance readings at OD = 600 nm. Error bars indicate standard deviations from three separate biological experiments

The growth of bacterial cells serves as a vital indicator of bacterial physiology [48]. In our study, we investigated the impact of heavy metal toxicity on the growth of *E.coli*-BL21(DE3)-pJET2.1-*CadA/CadR*-eGFP cells to gain insights into their behavior. We incubated the cells with varying concentrations of Cd^2+^ and monitored their growth by measuring absorbance at OD600 nm before conducting subsequent validation and calibration experiments. When comparing the growth curves of Cd^2+^ treated bacterial cells (with concentrations ranging from 1 to 6 ppb) with the growth curve of untreated cells, we observed no significant deviations. The growth followed the typical sigmoid pattern characteristic of bacterial growth. However, the 1 ppm treatment exhibited negligible growth, showing a severe impact of high heavy metal concentration on *E.coli*-BL21(DE3)-pJET2.1-*CadA/CadR*-eGFP cells (Fig. 3). These findings suggest that low heavy metal concentrations within the ppb range do not substantially affect the growth of our *E.coli*-BL21(DE3)-pJET2.1-*CadA/CadR*-eGFP cells, while a high concentration of 1 ppm severely hampers growth. Notably, heavy metals can exert toxicity on biological cells, including microorganisms [49]. Given that our microbial biosensor is designed to detect heavy metals in contaminated samples, understanding the adverse effects of corresponding heavy metals on bacterial cell growth and the ability to reconstruct the typical sigmoid growth curve in their presence provides crucial insights for our further study.

### *eGFP* mRNA expression analysis against heavy metal treatments

**Fig. 4:**
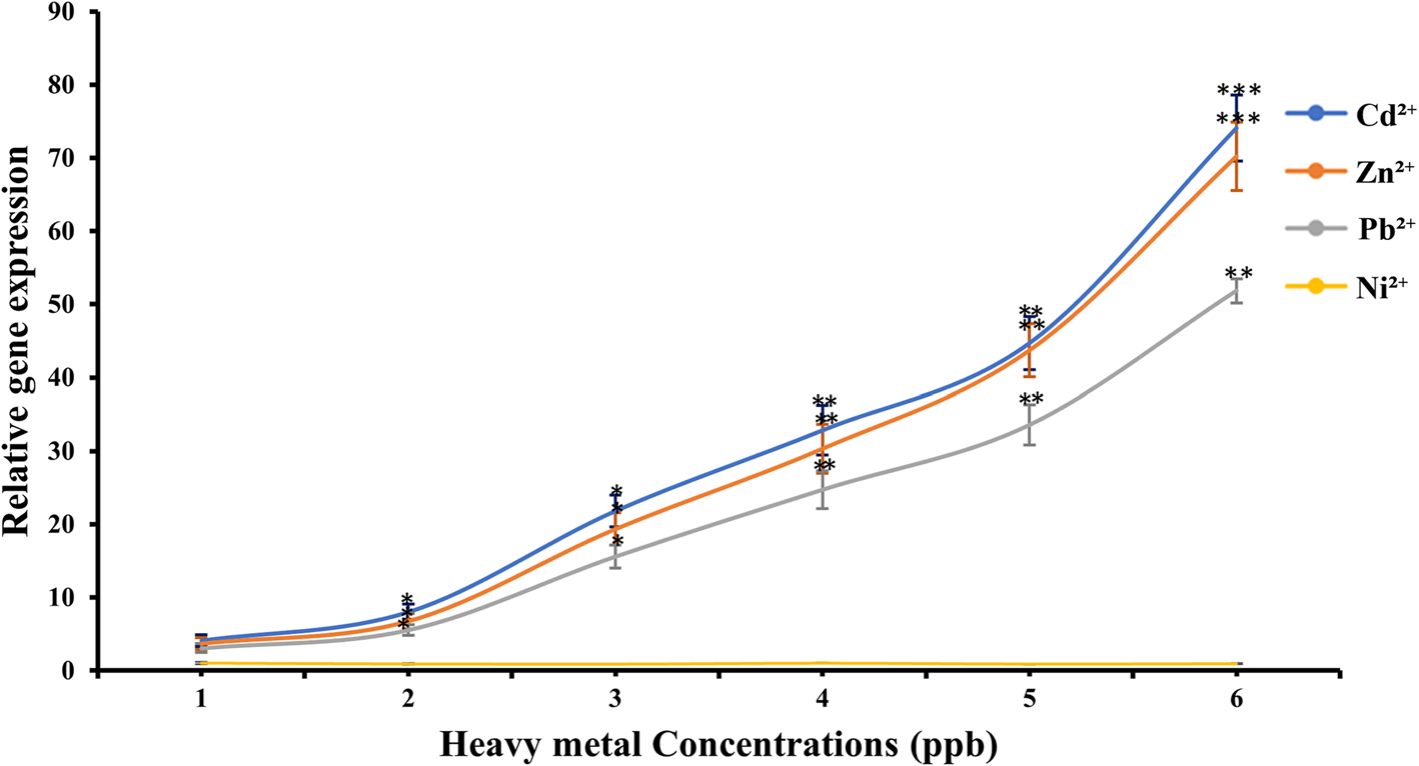
Relative gene expression of eGFP gene in response to Cd^2+^, Zn^2+^, Pb^2+^, and Ni^2+^ treatments. The plots depict the concentrations of Cd^2+^, Zn^2+^, Pb^2+^, and Ni^2+^ in blue, orange, ash, and yellow, respectively. The Y-axis represents the relative gene expression, while the X-axis denotes the metal ion concentration in ppb. Error bars indicate triplicate readings of relative gene expression for each heavy metal treatment. A single asterisk (*) represents a *P* value less than 0.05 (*P* < 0.05), indicating statistical significance at the 0.05 level. Double asterisks (**) represent a *P* value less than 0.01 (*P* < 0.01), indicating statistical significance at the 0.01 level. Triple asterisks (***) represent a *P* value less than 0.001 (*P* < 0.001), indicating statistical significance at the 0.001 level

The successfully transformed and growth-validated *E.coli*-BL21(DE3)-pJET1.2-*CadA/CadR* cells underwent qPCR testing to evaluate the expression of eGFP mRNA. Relative gene expression was plotted against varying heavy metal ion concentrations, yielding intriguing results. Notably, mRNA levels exhibited a significant increase in Cd^2+^, Zn^2+^, and Pb^2+^ treatments, whereas no substantial mRNA expression was observed in Ni^2+^ treatment. Statistical analysis using LSD confirmed the significance of Cd^2+^, Zn^2+^, and Pb^2+^ treatments with respect to elevated *eGFP* mRNA expression, beginning from the 2 ppb treatments compared to Ni^2+^. Particularly, *P* values reduced to 0.05 for 2 ppb and 3 ppb treatments, reaching even lower *P* values of 0.01 from 3 ppb treatment onwards for all three Cd^2+^, Zn^2+^, and Pb^2+^ treatments. For both Cd^2+^ and Zn^2+^, significance further increased at 6 ppb treatments, with *P* values dropping below 0.001 (Fig. 4). These gene expression outcomes signify the modified E.coli biosensor cells' ability to produce substantial eGFP mRNA upon exposure to specific heavy metals as per the genetic circuit. To the best of our knowledge, this study offers the first insight into assessing reporter mRNA loads of a genetically engineered bacterial biosensor for heavy metal analysis. The overall results demonstrate that the mRNA quantity produced in response to exposure to specific heavy metals is significantly higher than that produced in response to exposure to non-specific metal ions. Genes can serve as biomarkers, and gene expression can be integrated into biosensing applications for detecting various substances [50]. While direct assessment of gene expression through mRNA quantification has not been previously reported with microbial biosensors, it holds potential for future microbe-based biosensing applications.

### Fluorometry for eGFP protein expression analysis

**Fig. 5:**
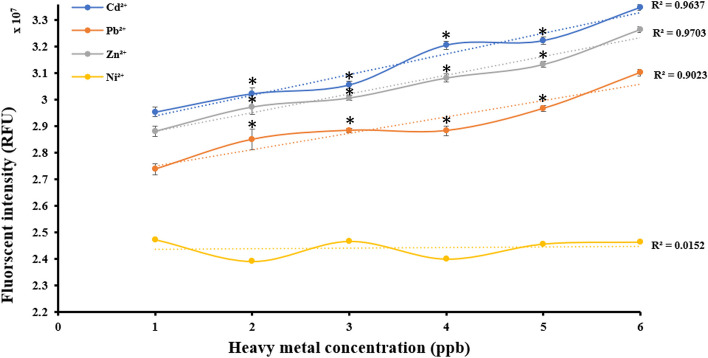
Fluorescent intensities (RFU) of *E.coli*-BL21-*CadA/CadR*-eGFP cells plotted against heavy metal treatment concentrations (ppb). The Y-axis represents the fluorescent intensity in RFU, while the X-axis represents the concentration of heavy metals in ppb. The plots for Cd^2+^, Pb^2+^, Zn^2+^, and Ni^2+^ are shown in blue, orange, ash, and yellow, respectively. The data were normalized to untreated *E.coli*-BL21-*CadA/CadR*-eGFP cells and wildtype *E.coli*-BL21. Error bars represent the standard error mean derived from three separate treatments. A single asterisk (*) represents a *P* value less than 0.05 (*P* < 0.05), indicating statistical significance at the 0.05 level. Double asterisks (**) represent a *P* value less than 0.01 (*P* < 0.01), indicating statistical significance at the 0.01 level. Triple asterisks (***) represent a *P* value less than 0.001 (*P* < 0.001), indicating statistical significance at the 0.001 level

In our study, we employed fluorometric analysis to determine the actual amount of eGFP proteins synthesized in response to heavy metal incubation, providing valuable insights at the protein level. The fluorometry approach allowed us to plot Fluorescent intensity in RFU (Relative Fluorescence Units) against Heavy metal concentrations, enabling a quantitative assessment of the correlation between fluorescent intensity and heavy metal concentration in ppb. Remarkably, we observed a linear correlation for all three specific metal ions, with R^2^ values of 0.9637, 0.9703, and 0.9023, respectively, for Cd^2+^, Zn^2+^, and Pb^2+^. Among these metal ions, Cd^2+^ exhibited the highest fluorescent signal intensity, followed by Zn2 + , while Pb^2+^ displayed the lowest intensity values. Starting from the 2 ppb concentration point, all treatment levels showed significantly elevated fluorescent signals (*P* < 0.05) for Cd^2+^, Zn^2+^, and Pb^2+^ compared to the non-specific Ni^2+^ treatment (Fig. 5). Our fluorometric findings collectively indicate that the *E.coli*-BL21-pJET2.1-*CadA/CadR*-eGFP cells effectively fluoresced specific signals in response to Cd^2+^, Zn^2+^, and Pb^2+^, exhibiting significant differentiation from the non-specific Ni^2+^ treatment. This aligns with a recent study conducted by Ravikumar's research group [29], demonstrating the capacity of a GFP and RFP dual reporter system to linearly and significantly respond to Ni^2+^ and Zn^2+^ in a genetically modified bacterial strain. These critical observations underscore the potential of *E.coli*-BL21-pJET2.1-*CadA/CadR*-eGFP cells for biosensing applications targeting specific heavy metals.

### Fluorescent microscopy to visualise eGFP based fluorescent signals

**Fig. 6:**
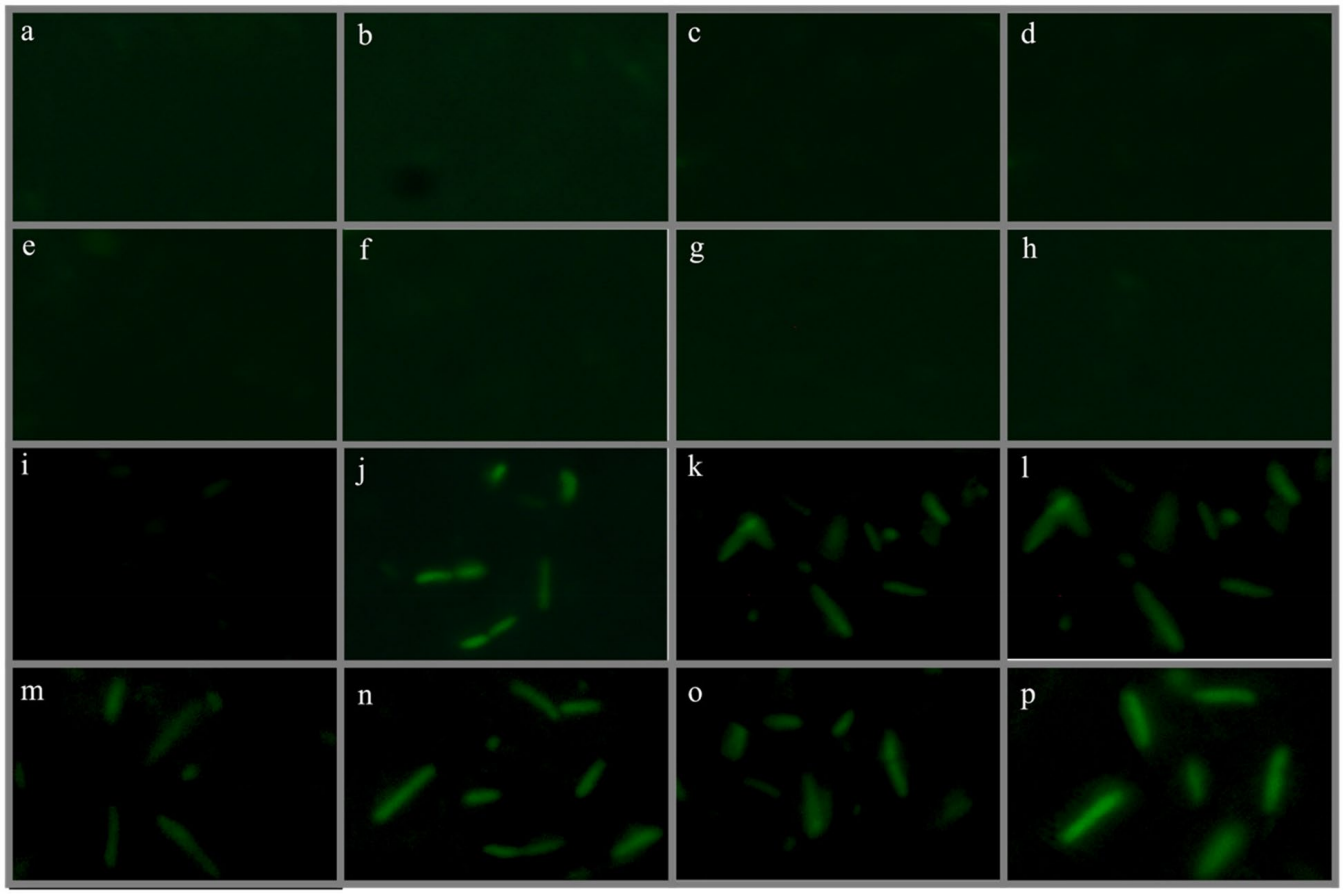
Fluorescent microscopic images of heavy metal-treated *E. coli*-BL21 cells. **a** Wildtype *E. coli*-BL21 cells with deionized water as the solvent. **b** Genetically modified *E.coli*-BL21-*CadA/CadR*-eGFP cells with deionized water as the solvent. **c** Untreated wildtype *E. coli*-BL21 cells regarding heavy metals. **d** Untreated genetically modified *E.coli*-BL21-*CadA/CadR*-eGFP cells regarding heavy metals. **e** Wildtype *E. coli*-BL21 cells treated with 2 ppb of Ni^2+^. **f** Wildtype *E. coli*-BL21 cells treated with 2 ppb of Cd^2+^. **g** Wildtype *E. coli*-BL21 cells treated with 2 ppb of Zn^2+^. **h** Wildtype *E. coli*-BL21 cells treated with 2 ppb of Pb^2+^. **i** Genetically modified *E.coli*-BL21-*CadA/CadR*-eGFP cells treated with 2 ppb of Ni^2+^. **j** Genetically modified *E.coli*-BL21-*CadA/CadR*-eGFP cells treated with 2 ppb of Cd^2+^. **k** Genetically modified *E.coli*-BL21-*CadA/CadR*-eGFP cells treated with 2 ppb of Zn^2+^. **l** Genetically modified *E.coli*-BL21-*CadA/CadR*-eGFP cells treated with 2 ppb of Pb^2+^. **m** Genetically modified *E.coli*-BL21-*CadA/CadR*-eGFP cells treated with 2 ppb of Cd^2+^ and Pb^2+^. **n** Genetically modified *E.coli*-BL21-*CadA/CadR*-eGFP cells treated with 2 ppb of Cd^2+^ and Zn^2+^. **o** Genetically modified *E.coli*-BL21-*CadA/CadR*-eGFP cells treated with 2 ppb of Pb^2+^ and Zn^2+^. **p** Genetically modified *E.coli*-BL21-*CadA/CadR*-eGFP cells treated with 2 ppb of Cd^2+^, Zn^2+^, and Pb.^2+^. (FITC filter was used for all images, and the magnification was set at × 600)

In our fluorescent microscopy experiment, we utilized this technique to capture images of heavy metal-treated *E.coli*-BL21-pJET2.1-*CadA/CadR*-eGFP cells for subsequent analysis using image processing tools. In the initial rounds of image capturing, we observed visible fluorescent signals in all *E.coli*-BL21-pJET2.1-*CadA/CadR*-eGFP cells subjected to Cd^2+^, Pb^2+^, Zn^2+^, (Cd^2+^ and Pb^2+^ mixture), (Cd^2+^ and Zn^2+^ mixture), (Pb^2+^ and Zn^2+^ mixture), and (Cd^2+^, Zn^2+^, and Pb^2+^ mixture) treatments under the fluorescent microscope (Fig. 6). Conversely, the control treatments did not exhibit any detectable fluorescent signals in the microscopic images. These microscopic imaging results further confirmed that the genetically modified biosensing cells successfully expressed the eGFP proteins in response to the exposure to specific heavy metals, as designed in our genetic circuit. This aligns with findings from a prior study where a Green Fluorescent Protein reporter-based bacterial biosensor effectively detected Cd, Pb, and Sb bioavailability, showing intensified fluorescent signals with a CCD camera at × 1000 magnification [6]. Additionally, a recent study by Ravikumar [29] demonstrated dual-color overlays and single-color microscopic images with intense fluorescence signals upon exposure to 0.1 mM Zn^2+^, Cu^2+^, and a combination of these metals. The GFP and Red Fluorescent Protein (RFP) reporters in this study simultaneously responded to different heavy metal ions and were detected using an Olympus reflected fluorescence microscope equipped with a Peltier-cooled CCD camera.

Our results, together with these previous findings, highlight the effectiveness of fluorescent microscopy in visualizing and validating the expression of specific proteins in genetically modified biosensing cells under heavy metal exposure. This technique offers valuable insights into the response of biosensors to heavy metal mixtures, further enhancing our understanding of their potential applications in environmental monitoring and detection.

### The physiology of *E.coli*-BL21(DE3)-pJET1.2-*CadA/CadR* cells

Bacterial cells, as microorganisms, are highly influenced by environmental conditions such as temperature, pH, and nutrient levels in the medium [51]. For biosensing applications employing bacterial cells as host organisms, it is crucial to validate their physiological parameters, including optimal temperature, pH of growth medium, nutritional requirements, and tolerance to toxic substances. In this study, we conducted physiological validation experiments on modified *E.coli*-BL21-pJET2.1-*CadA/CadR*-eGFP cells to determine their applicational range.

To support the growth of bacterial cells, LB broth and LB agar media were used throughout the experiments [52]. Additionally, we evaluated the ability of *E.coli*-BL21-pJET2.1-*CadA/CadR*-eGFP cells to emit significant fluorescent signals under optimal *E.coli* bacteria growth conditions, i.e., at a pH level of approximately 7.0 and a temperature of 37 °C.

#### eGFP fluorescent signals under optimum pH conditions

**Fig. 7:**
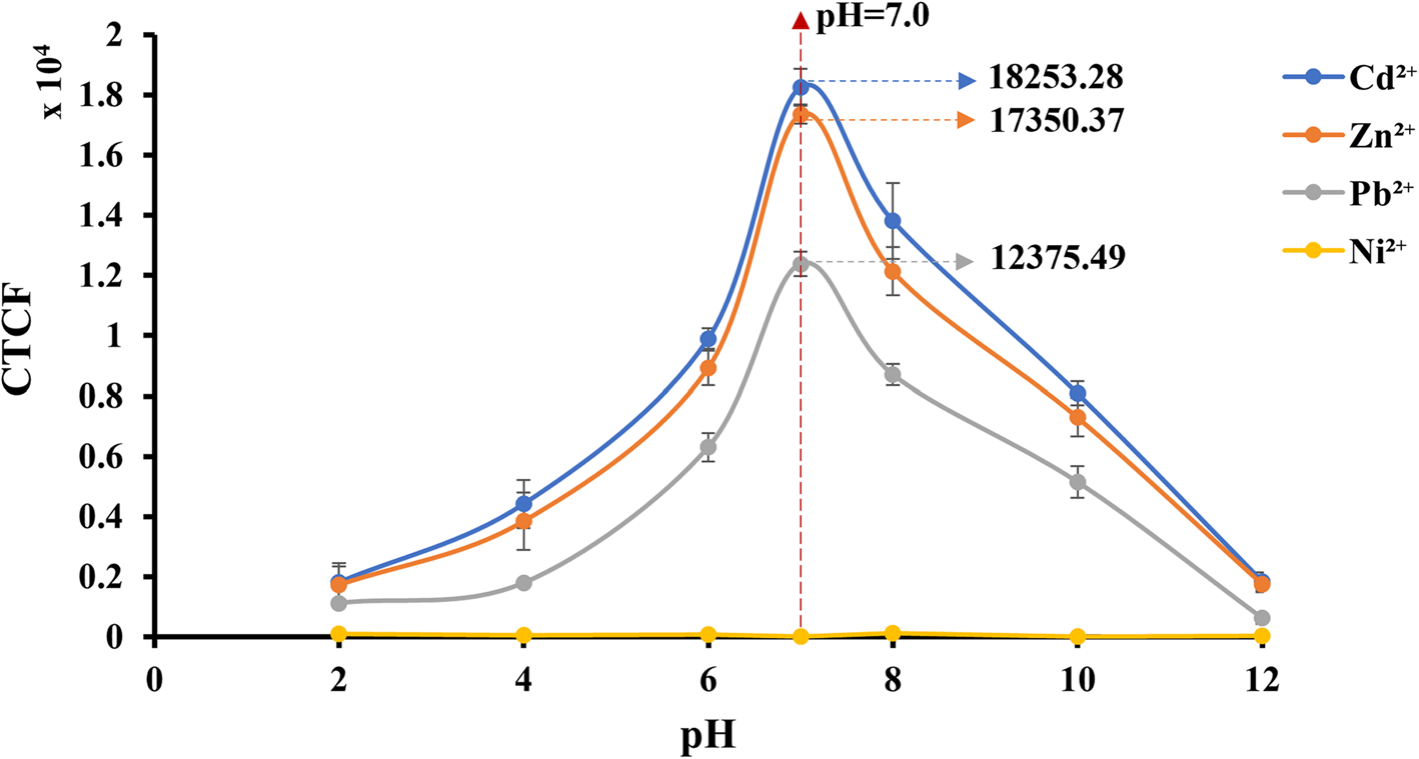
Normalized CTCF values plotted against pH. The Y-axis represents fluorescent intensities in CTCF, while the X-axis represents pH values ranging from 2 to 12. Error bars indicate the mean standard deviation derived from three independent biological treatments. The cells were treated with 2.0 ppb of Cd^2+^, Zn^2+^, Pb^2+^, and Ni^2+^. The cells were grown at a constant temperature of 37 °C. Plots for Cd^2+^, Zn^2+^, Pb^2+^, and Ni^2+^ are depicted in blue, orange, ash and yellow, respectively, in the specified order

Ideally, wildtype bacterial cells exhibit their optimal physiological activities under specific pH levels of the growth medium. For *E.coli* cells, the optimal growth pH condition is shown to be 5.5–7.0 [53]. These physiological alterations can, in turn, affect gene expression patterns, and even gene modifications can have specific impacts on bacterial physiology. Therefore, the intensity of fluorescent signals emitted from *E.coli*-BL21-pJET2.1-*CadA/CadR*-eGFP cells was examined across a series of pH values (2, 4, 6, 8, 10, and 12), recording intensity values in CTCF for Cd^2+^, Zn^2+^, and Pb^2+^ as 18253.28, 17350.37, and 12375.49, respectively (Fig. 7). Notably, these values were significantly elevated at pH 5.5–7.0, demonstrating the importance of maintaining this pH range for subsequent biosensing cell growth in any heavy metal detection application utilizing *E.coli*-BL21-pJET2.1-*CadA/CadR*-eGFP cells.

#### eGFP fluorescent signals in optimum temperature range

The cellular processes within living organisms, including crucial functions like protein folding and enzyme activation, are profoundly affected by fluctuations in temperature [54]. For *E. coli* bacterial cells, the optimal growth temperature for maintaining natural physiological conditions is recognized to be 37 °C [55]. In our research, we examined the performance of *E. coli*-BL21-pJET2.1-*CadA/CadR*-eGFP cells in generating robust green fluorescent signals across a range of temperatures that impact *E. coli* physiology.

**Fig. 8:**
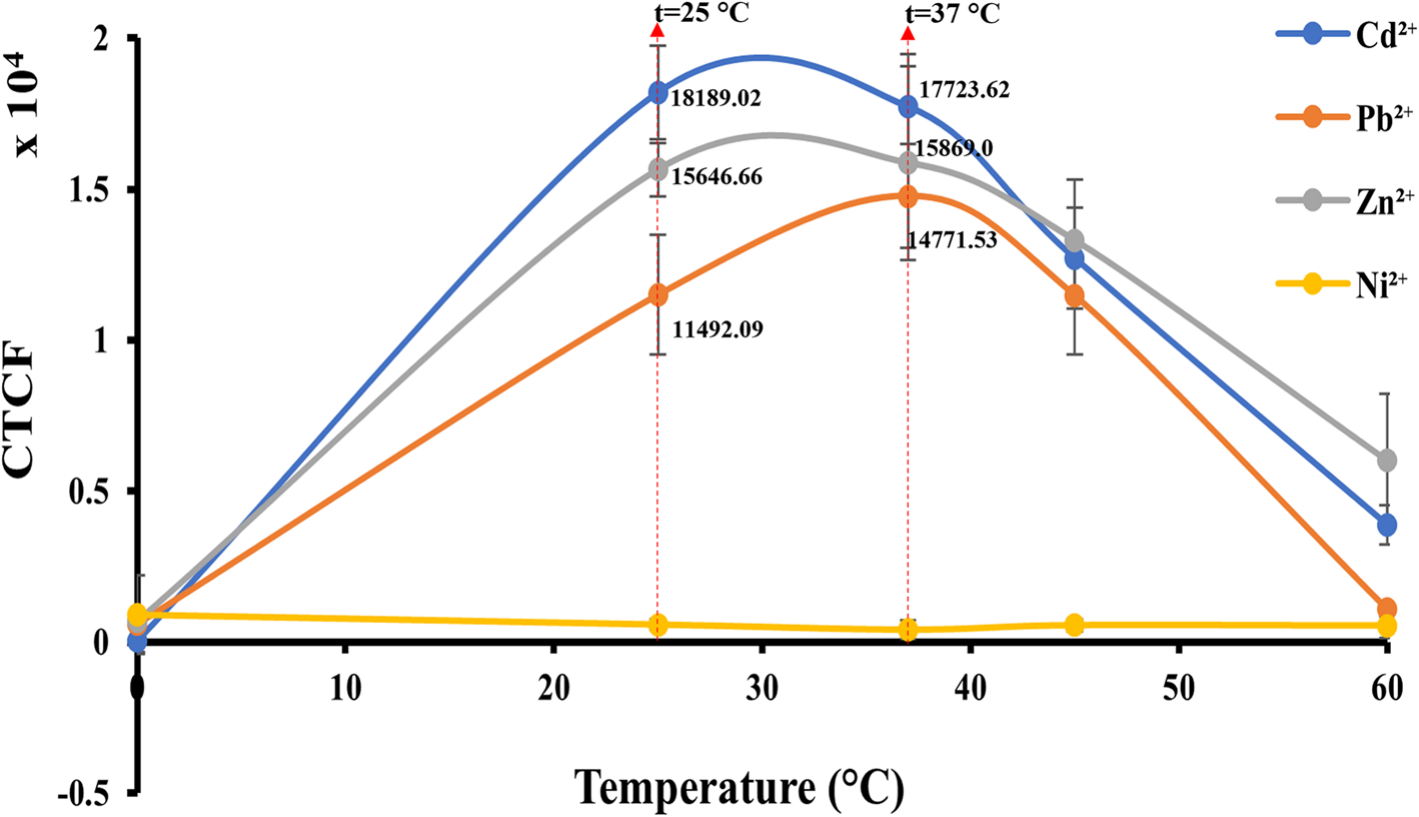
Normalized CTCF values plotted against incubation temperature. The Y-axis represents fluorescent intensities in CTCF, while the X-axis represents incubation temperature in °C. Error bars indicate the standard deviation derived from three independent biological treatments. The cells were treated with 2.0 ppb of Cd^2+^, Zn^2+^, Pb^2+^, and Ni^2+^. The cells were grown at a constant pH of 7.0. Plots for Cd^2+^, Zn^2+^, Pb^2+^, and Ni^2+^ are depicted in blue, ash, orange, and yellow, respectively, in the specified order

Upon subjecting these cells to treatments with Cd^2+^, Pb^2+^, and Zn^2+^, we observed distinct responses at different temperatures. Specifically, at 25 °C, the CTCF value for Cd^2+^ was measured at 18189.02, while at 37 °C, the CTCF value for Cd^2+^ was slightly lower at 17723.62. Intermediate temperatures yielded CTCF values that were marginally higher. For Zn^2+^, the CTCF value at 25 °C was 15646.66, whereas at 37 °C, it rose to 15869.0, with intermediate temperatures showing slightly elevated values. Finally, for Pb^2+^, the CTCF value at 25 °C was 11492.09, but it reached its peak at 37 °C with a CTCF value of 14771.53. Notably, all these CTCF values, for all three metals tested at 25 °C, 37 °C, and intermediate temperatures, exhibited a significant increase compared to the values at 0 °C (Fig. 8).

Taken together, our analysis of eGFP signals at different temperatures underscores the versatility of *E. coli*-BL21-pJET2.1-*CadA/CadR*-eGFP cells for applications involving the fluorescent detection of heavy metals. These cells perform effectively not only at the physiologically crucial 37 °C but also at room temperature (25 °C), making them valuable for various environmental and biological assays.

#### Effect of heavy metal incubation time on the eGFP fluorescent signals

To activate the heavy metal-sensitive genetic circuit in bacterial cells, the presence of exogenous metal ions is essential to interact with the promoter and initiate mRNA expression of the gene circuit. This metal absorption process requires a specific incubation time with the sample. To investigate the relationship between incubation time and fluorescent signals, we analyzed the patterns of signal generation for Cd^2+^, Zn^2+^, and Pb^2+^ at different time points and concentrations.

**Fig. 9:**
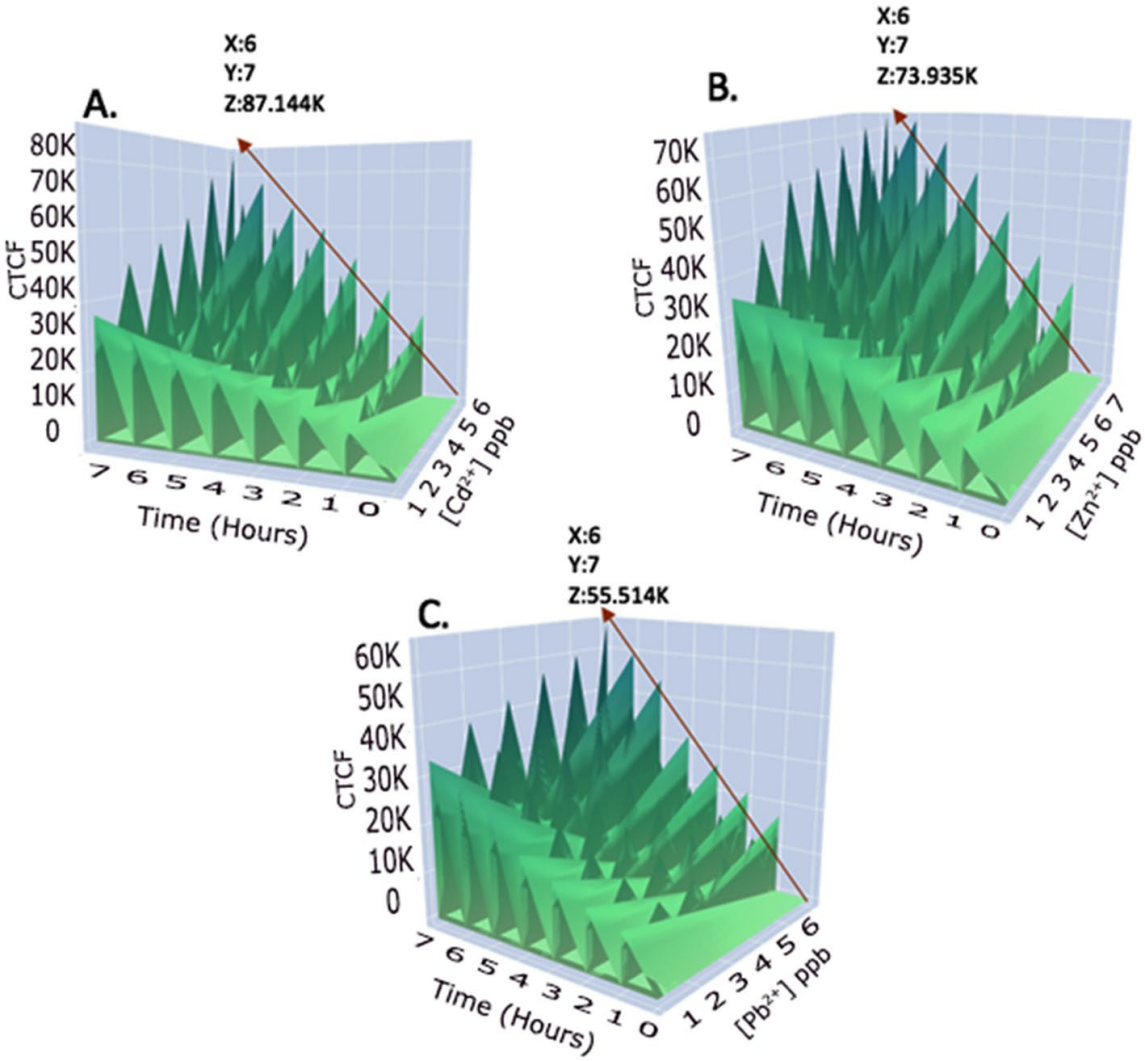
Time-dependent fluorescent responses of *E.coli*-BL21-pJET2.1-*CadA/CadR*-eGFP cells. The X-axis represents time, the Y-axis represents heavy metal concentration, and the Z-axis represents fluorescent intensities in CTCF. The gradual increment of CTCF signal is indicated by the red arrow. **A** Cd^2+^ treatment plot. **B** Zn^2+^ treatment plot. **C** Pb^2+^ treatment plot

We represented the fluorescent signals in CTCF values against heavy metal concentrations and incubation time on the same graph, with concentration on the X-axis, incubation time on the Y-axis, and fluorescent intensity in CTCF on the Z-axis. Interestingly, Cd^2+^, Zn^2+^, and Pb^2+^ demonstrated their highest fluorescent intensity values when incubation time was the longest. For instance, Cd^2+^ at 7 h incubation and 6 ppb concentration exhibited a CTCF intensity of 87.144 K (Fig. 9A), while Zn^2+^ and Pb^2+^ showed CTCF intensities of 73.935 K (Fig. 9B) and 55.514 K (Fig. 9C), respectively. Furthermore, we observed a gradual increase in CTCF signals for all three metal ions with prolonged incubation time. This trend has been consistent in other microbial biosensor studies as well. Previous research, such as Stocker's Arsenate/Arsanite detecting bacterial biosensor [56] and Liao's GFP-based biosensor [6] for detecting heavy metal bioavailability in soil, has reported similar patterns. These findings indicate the importance of incubation time in achieving optimal fluorescent signals, supporting the effectiveness of our bacterial biosensor in detecting heavy metals.

### Calibration of biosensor

#### Concentration dependent responses

##### Individual heavy metal treatments

To understand the ability of *E. coli*-BL21-pJET2.1-*CadA/CadR*-eGFP cells to generate specific and consistent fluorescent signals against corresponding heavy metals, the R^2^ values were analysed on a plot between total heavy metal concentration in ppb and green fluorescent signal intensities in CTCF.

**Fig. 10:**
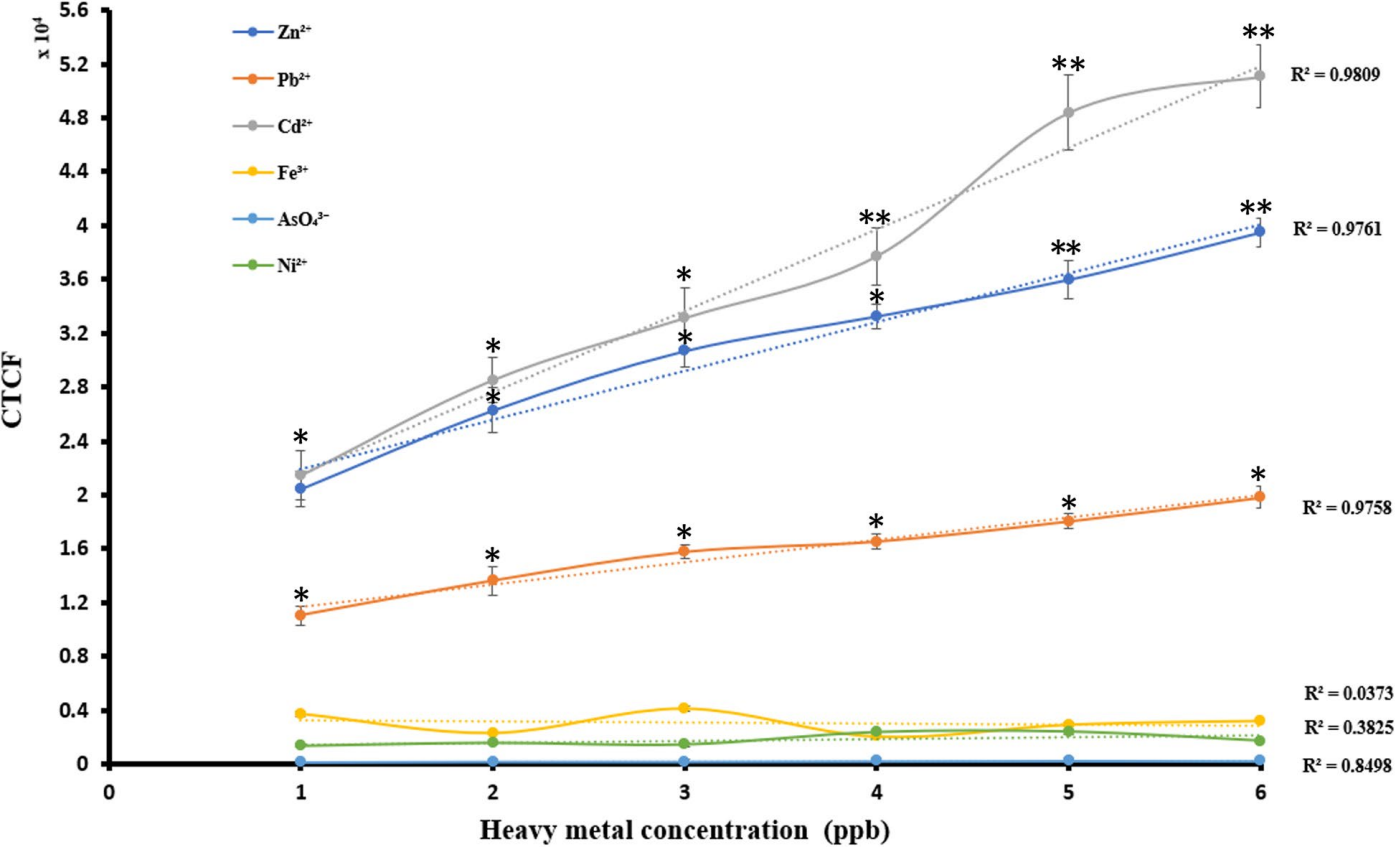
Calibration plot of heavy metal treatments correlating fluorescent intensities in CTCF with heavy metal concentrations in ppb. The plot encompasses metal ion concentrations ranging from 1 to 6 ppb. Cd^2+^, Zn^2+^, Pb^2+^, Ni^2+^, Fe^3+^ and AsO_4_^3−^ are represented by the colours Ash, Blue, Orange, Green, Yellow and Light Blue, respectively. Each plot illustrates the CTCF variation corresponding to the metal ion concentration. Dotted lines indicate trendlines for each metal ion, along with their respective R^2^ values. Error bars represent the standard mean differences derived from three independent biological treatments. A single asterisk (*) represents a *P* value less than 0.05 (*P* < 0.05), indicating statistical significance at the 0.05 level. Double asterisks (**) represent a *P* value less than 0.01 (*P* < 0.01), indicating statistical significance at the 0.01 level. Triple asterisks (***) represent a *P* value less than 0.001 (*P* < 0.001), indicating statistical significance at the 0.001 level

The calibration of the *E.coli*-BL21-pJET2.1-*CadA/CadR*-eGFP cells biosensor against specific heavy metal treatments (Cd^2+^, Zn^2+^, and Pb^2+^) and non-specific heavy metals (Ni^2+^, Fe^3+^, and AsO_4_^3−^) revealed significant and promising findings. Fluorescent signals plotted against heavy metal concentrations (in ppb) showed robust linear correlations for Cd^2+^ (R2 = 0.9809), Zn^2+^ (R2 = 0.9761), and Pb^2+^ (R2 = 0.9758), indicating a reliable response to these specific heavy metals. In contrast, non-specific ions Ni^2+^, Fe^3+^, and AsO_4_^3−^ exhibited lower R^2^ values of 0.3825, 0.0373, and 0.8498, respectively, suggesting a weaker response to these metals. Furthermore, the biosensor demonstrated significantly elevated (*P* < 0.05) fluorescent signal levels for Cd^2+^, Zn^2+^, and Pb^2+^ compared to non-specific ions across all treatment points (1–6 ppb). Conversely, the signals obtained for non-specific Ni^2+^, Fe^3+^, and AsO_4_^3−^ were negligible, confirming the biosensor's specificity to the target heavy metals (Fig. 10).

**Fig. 11:**
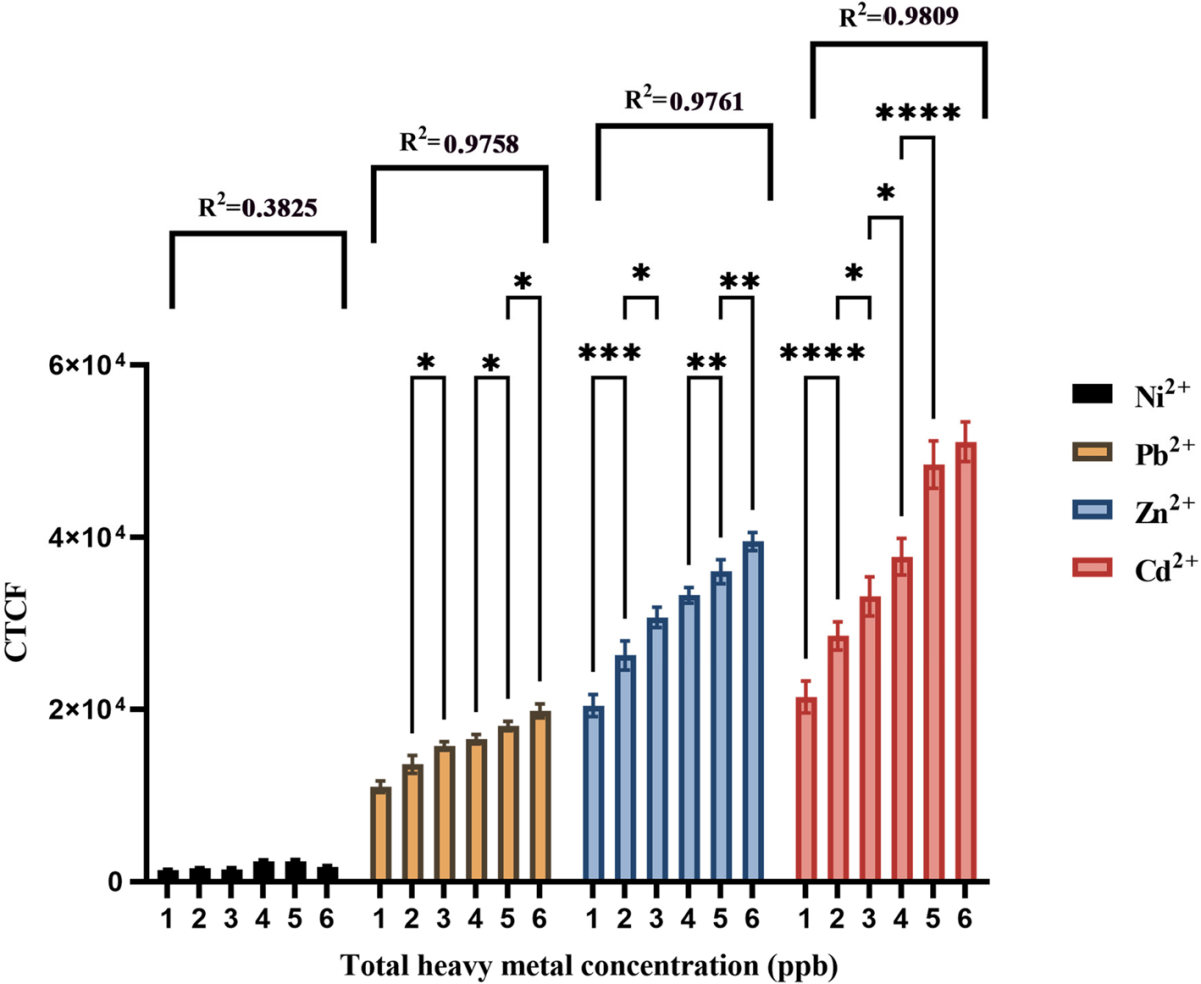
Calibration plot of individual heavy metal treatments between fluorescent intensities in CTCF and heavy metal concentrations in ppb. The fluorescent intensities have been obtained for samples containing metal ion concentrations in between 1 to 6 ppb. The treatments with Ni^2+^, Pb^2+^, Zn^2+^ and Cd^2+^ are shown in black colour bars, orange colour bars, blue colour bars and red colour bars in order. The R^2^ values for Ni^2+^, Pb^2+^, Zn^2+^ and Cd^2+^ trends are as 0.3825, 0.9785, 0.9761 and 0.9809. The error bars represent standard mean differences of three independent biological treatments. A single asterisk (*) represents a *P* value less than 0.05 (*P* < 0.05), indicating statistical significance at the 0.05 level. Double asterisks (**) represent a *P* value less than 0.01 (*P* < 0.01), indicating statistical significance at the 0.01 level. Triple asterisks (***) represent a *P* value less than 0.001 (*P* < 0.001), indicating statistical significance at the 0.001 level. Quadruple asterisks (****) represent a *P* value less than 0.0001 (*P* < 0.0001), indicating statistical significance at the 0.0001 level

Bar chart analysis revealed distinct signal resolutions for each specific metal, with Cd^2+^ showing significant resolution between 1–2 ppb (*P* < 0.0001), 2–3 ppb (*P* < 0.05), 3–4 ppb (*P* < 0.05), and 4–5 ppb (*P* < 0.0001), and Zn^2+^ displaying significant resolution between 1–2 ppb (*P* < 0.001) and maintaining significance between 4–5 ppb and 5–6 ppb (*P* < 0.01). In contrast, Pb^2+^ showed significant resolutions between 2–3 ppb, 3–4 ppb, 4–5 ppb, and 5–6 ppb signal levels. Notably, no significant signal resolution was observed for Ni^2+^ across any treatments from 1 to 6 ppb (Fig. 11). Overall, these results highlight the biosensor's high specificity for Cd^2+^, Zn^2+^ and Pb^2+^ detection, which could be attributed to the distinct chemical properties and variations in binding capacities of the metals to the pJET1.2-*CadA/CadR* gene construct promoter region. The findings demonstrate the potential of the *E.coli*-BL21-pJET2.1-*CadA/CadR*-eGFP cells biosensor as a reliable tool for environmental toxicology and biosensing applications. The higher specificity of Cd^2+^ and Zn^2+^ compared to Pb^2+^ has been observed and this can corelate to the different chemical properties of the metals and the variations of their binding capacities to the promoter region of the pJET1.2-CadA/CadR gene construct [57]. The observed higher specificity of Cd^2+^ and Zn^2+^ in comparison to Pb^2+^ may be attributed to the distinct chemical properties of these metals and their variations in binding capacities to the promoter region of the pJET1.2-*CadA/CadR* gene construct [57].

##### Heavy metal mixture treatments

In general, treatments involving mixtures of heavy metals exhibit a characteristic tendency to competitively interact with gene circuits due to similarities and dissimilarities in their chemical properties.

**Fig. 12:**
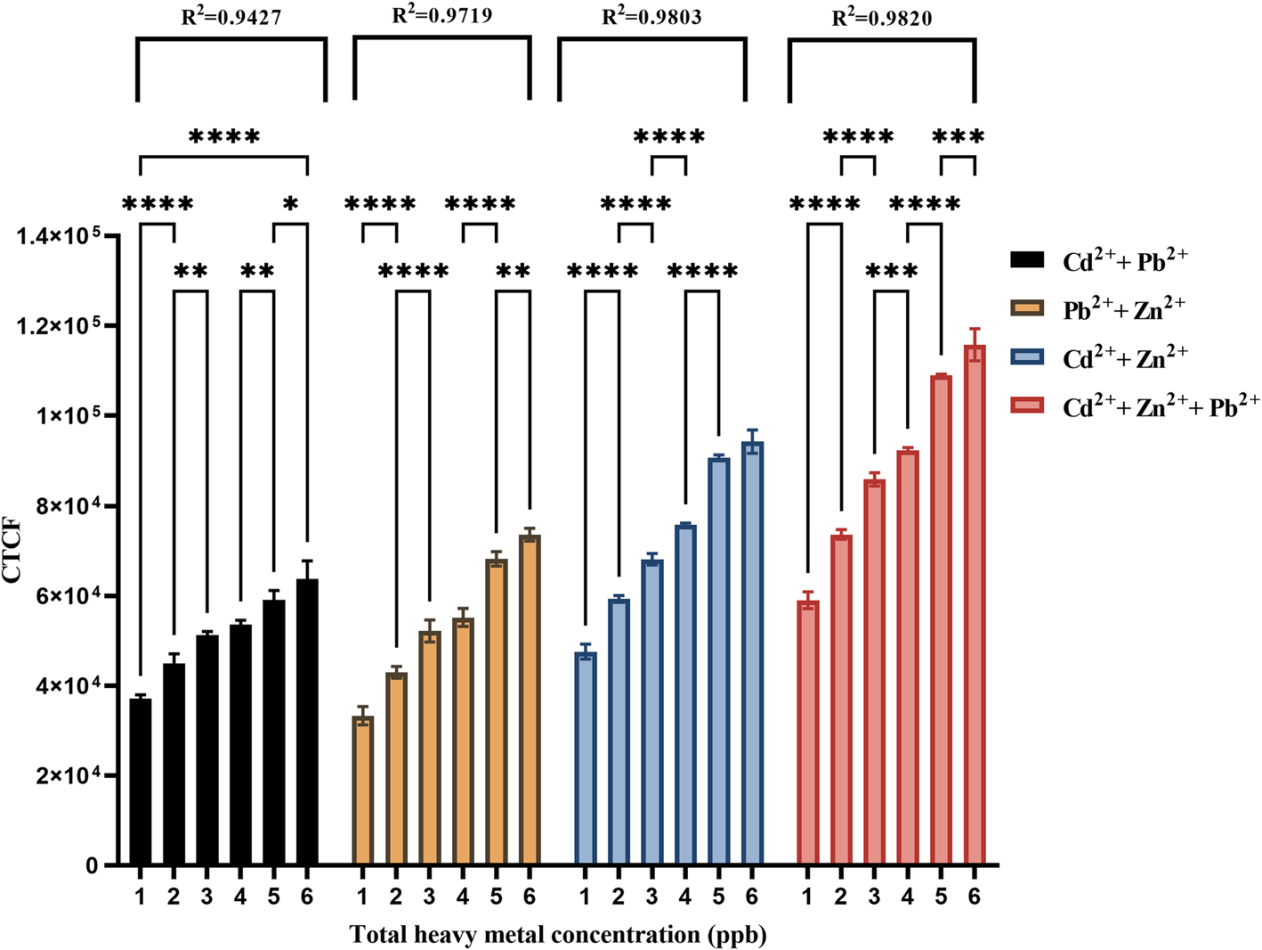
Calibration plot of heavy metal combination treatments between fluorescent intensities in CTCF and heavy metal concentrations in ppb. The fluorescent intensities have been obtained for samples containing metal ion concentrations in between 1 to 6 ppb of total heavy metal concentrations. The treatments with [Cd^2+^  + Pb^2+^], [Pb^2+^ + Zn^2+^], [Cd^2+^  + Zn^2+^], and [Cd^2+^  + Zn^2+^  + Pb^2+^] are shown in black colour bars, orange colour bars, blue colour bars and red colour bars in order. The R^2^ values for [Cd^2+^  + Pb^2+^], [Pb^2+^ + Zn^2+^], [Cd^2+^  + Zn^2+^], and [Cd^2+^  + Zn^2+^  + Pb^2+^] combination treatment trends are as 0.9427, 0.9719, 0.9803 and 0.9820. The error bars represent standard mean differences of three independent biological treatments. A single asterisk (*) represents a *P* value less than 0.05 (*P* < 0.05), indicating statistical significance at the 0.05 level. Double asterisks (**) represent a *P* value less than 0.01 (*P* < 0.01), indicating statistical significance at the 0.01 level. Triple asterisks (***) represent a *P* value less than 0.001 (*P* < 0.001), indicating statistical significance at the 0.001 level. Quadruple asterisks (****) represent a *P* value less than 0.001 (*P* < 0.0001), indicating statistical significance at the 0.0001 level

**Fig. 13:**
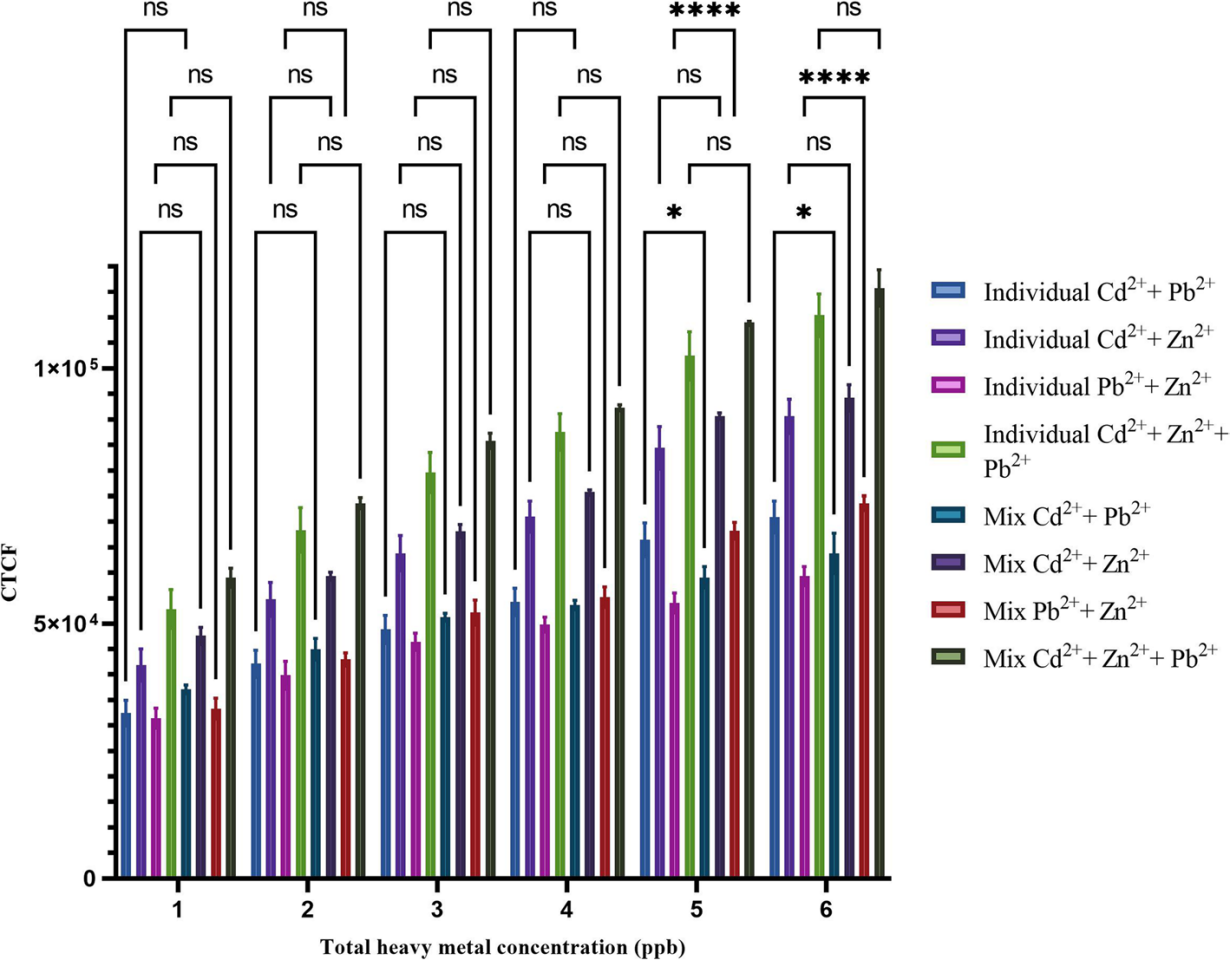
The comparative analysis of fluorescent intensities in CTCF for heavy metal mixture treatments and individual heavy treatments. The fluorescent intensities obtained for individual heavy metal treatments of Cd^2+^  + Pb^2+^, Pb^2+^ + Zn^2+^, Cd^2+^  + Zn^2+^, and Cd^2+^  + Zn^2+^  + Pb^2+^are shown in light blue colour bars, light purple colour bars, light pink colour bars and light green colour bars in order. The fluorescent intensities obtained for heavy metal mixture treatments of [Cd^2+^  + Pb^2+^], [Pb^2+^ + Zn^2+^], [Cd^2+^  + Zn^2+^], and [Cd^2+^  + Zn^2+^  + Pb^2+^ are shown in dark blue colour bars, dark purple colour bars, dark pink colour bars and dark green colour bars in order. The error bars represent standard mean differences of three independent biological treatments. A single asterisk (*) represents a *P* value less than 0.05 (*P* < 0.05), indicating statistical significance at the 0.05 level. Double asterisks (**) represent a *P* value less than 0.01 (*P* < 0.01), indicating statistical significance at the 0.01 level. Triple asterisks (***) represent a *P* value less than 0.001 (*P* < 0.001), indicating statistical significance at the 0.001 level. Quadruple asterisks (****) represent a *P* value less than 0.001 (*P* < 0.0001), indicating statistical significance at the 0.0001 level

In our study, we investigated the interactions of Cd^2+^, Zn^2+^, and Pb^2+^ with the *CadA/CadR*-eGFP genetic circuit by combining these heavy metals as mixtures for subsequent treatments. The [Cd^2+^  + Pb^2+^] mixture displayed an increasing trend with an R^2^ value of 0.9427. The differences between treatments were statistically significant, with a significance level of *P* < 0.05 for each treatment point. Similarly, the [Pb^2+^  + Zn^2+^] mixture exhibited an R^2^ value of 0.9719, and the significance levels between treatment points were detected to be at least *P* < 0.01 or lower. For the [Cd^2+^  + Zn^2+^] combination, the R^2^ value was 0.9803, and the treatment resolution was observed to be significant with *P* < 0.0001 for any two adjacent treatments. Finally, when all three metals were combined [Cd^2+^  + Zn^2+^  + Pb^2+^], the R^2^ value was 0.9820, and the treatment resolution remained significant with at least *P* < 0.001 or below for any two adjacent treatments (Fig. 12). When comparing the CTCF signals obtained for heavy metal mixtures to those of individual heavy metal treatments, we observed that mixtures containing [Cd^2+^  + Pb^2+^], [Pb^2+^  + Zn^2+^], [Cd^2+^  + Zn^2+^], and [Cd^2+^  + Zn^2+^  + Pb^2+^] showed no significant differences at concentrations of 1 ppb, 2 ppb, 3 ppb, and 4 ppb. However, at higher concentrations of 5 ppb and 6 ppb, some mixture treatments exhibited significant differences compared to individual treatments. Specifically, the [Cd^2+^  + Pb^2+^] treatment at 5 ppb showed significance with a *p* value < 0.05, while the [Pb^2+^  + Zn^2+^] treatment at 5 ppb demonstrated high significance with a *p* value < 0.0001. Similarly, the [Cd^2+^  + Pb^2+^] treatment at 6 ppb showed significance with a *p* value < 0.05, and the [Pb^2+^  + Zn^2+^] treatment at 6 ppb exhibited high significance with a *p* value < 0.0001 (Fig. 13).

The intricate interactions of multiple metal ions within a single gene circuit present challenges when conducting biosensing assays on samples containing mixtures of heavy metals. The noteworthy changes observed at concentrations of 5 ppb and 6 ppb for [Cd^2+^  + Pb^2+^] and [Pb^2+^  + Zn^2+^] treatments may be attributed to the competitive affinities of these slightly different heavy metals for distinct transcription factors at a molecular level. This intriguing phenomenon warrants further investigation before deploying the biosensor for heavy metal detection in mixtures. However, mathematical modelling approaches can provide a solution for quantifying individual metal ion concentrations within a mixture using a single reporter gene and curve-fitting equations. Alternatively, employing dual reporter molecules with separate promoters can mitigate the combined effects when analyzing mixtures, as demonstrated by Ravikumar, who utilized two distinct reporter genes and promoters [29]. In this study, the reporter genes emitted fluorescence signals at different wavelengths, allowing for their separate identification or as a colour overlay.

## Conclusion

In current study, we successfully constructed a genetically engineered microbial biosensor using the *CadA/CadR*-eGFP genetic circuit in *E. coli*-BL21(DE3) cells for the detection of Cd^2+^, Zn^2+^ and Pb^2+^ heavy metal ions. The selected genetic circuit, based on the *CadA/CadR* operon from *Pseudomonas aeruginosa*, demonstrated specific response to these heavy metals, as supported by previous studies. The biosensor's functioning relied on a NOT molecular logic gate, where the expression of the reporter gene (*eGFP*) was induced in the presence of the target metal ions and terminated in their absence. The use of a *T7* promoter system in the plasmid vector facilitated efficient transcription of the CadR gene, and subsequent interactions with Cd^2+^, Pb2^+^, and Zn^2+^ induced the expression of fluorescent signals corresponding to the metal concentrations.

Physiological validation experiments demonstrated that the *E.coli*-BL21(DE3)-pJET2.1-*CadA/CadR*-eGFP cells grew and fluoresced optimally at pH 5.5–7.0 and at a temperature of 37 °C. Furthermore, incubation time played a crucial role in achieving maximum fluorescent signals, as prolonged incubation resulted in higher intensity values. Fluorometric analysis showed strong linear correlations between fluorescent intensity and heavy metal concentrations for Cd^2+^, Zn^2+^, and Pb^2+^, with Cd^2+^ exhibiting the highest fluorescent signal intensity.

The calibration of the biosensor against individual heavy metal treatments revealed robust linear correlations for Cd^2+^, Zn^2+^, and Pb^2+^ and negligible responses to non-specific heavy metal ions (Ni^2+^, Fe^3+^, and AsO_4_^3−^). The biosensor demonstrated significantly elevated fluorescent signal levels for Cd^2+^, Zn^2+^, and Pb^2+^ compared to non-specific ions, and the signal resolutions were distinct for each specific metal.

Furthermore, the biosensor was validated for its ability to detect heavy metal mixtures. The interactions of Cd^2+^, Zn^2+^, and Pb^2+^ with the genetic circuit when combined as mixtures showed significant trends and resolutions between different treatments. Although the synergetic interactions of multiple metal ions pose challenges in biosensing operations for samples containing heavy metal mixtures, mathematical model approaches and the use of dual reporter molecules with separate promoters can address these concerns.

Collectively, our genetically engineered microbial biosensor, *E. coli*-BL21(DE3)-pJET2.1-*CadA/CadR*-eGFP, demonstrated reliable and specific detection capabilities for Cd^2+^, Zn^2+^, and Pb^2+^ heavy metal ions. Its robust linear responses, distinct signal resolutions, and high specificity make it a promising tool for environmental toxicity assessment and biosensing applications targeting specific heavy metals. The research findings contribute to the field of microbial biosensors and offer valuable insights for future applications in environmental monitoring and detection of heavy metal contaminants.

### Supplementary Information

**Additional file 1:** **Supplementary figure 01.** Agarose gel electrophoresis of colony PCR products of *E.coli*-BL21-pJET1.2-*CadA/CadR* cells (Modified and cropped image) Well Numbers: M- 100bp DNA ladder, 2- positive control DNA construct, 3-9 - isolated colonies of *E.coli*-BL21-pJET1.2-*CadA/CadR* cells. **Supplementary figure 02.** An uncropped image of agarose gel electrophoresis of colony PCR products of *E. coli*-BL21-pJET1.2-*CadA/CadR* cells Well Numbers: 1- 100bp DNA ladder, 2- positive control DNA construct, 3-9 - isolated colonies of  *E.coli*-BL21-pJET1.2-*CadA/CadR* cells.

## Data Availability

The standard data sets analysed during the current study were included in the manuscript. Additional data or materials can be obtained from the corresponding author upon request.
