# Supplementary material for: Validation and calibration of a novel GEM biosensor for specific detection of Cd2+, Zn2+, and Pb2+
Source: BMC Biotechnol. 2023 Dec 8;23:52. doi: 10.1186/s12896-023-00820-7 (PMC10709830; doi:10.1186/s12896-023-00820-7)
Supplement: Supplementary file 1 — Additional file 1: Supplementary figure 01. Agarose gel electrophoresis of colony PCR products of E.coli-BL21-pJET1.2-CadA/CadR cells (Modified and cropped image) Well Numbers: M- 100bp DNA ladder, 2- positive control DNA construct, 3-9 - isolated colonies of E.coli-BL21-pJET1.2-CadA/CadR cells. Supplementary figure 02. An uncropped image of agarose gel electrophoresis of colony PCR products of E. coli-BL21-pJET1.2-CadA/CadR cells Well Numbers: 1- 100bp DNA ladder, 2- positive control DNA construct, 3-9 - isolated colonies of E.coli-BL21-pJET1.2-CadA/CadR cells. [file 12896_2023_820_MOESM1_ESM.docx]

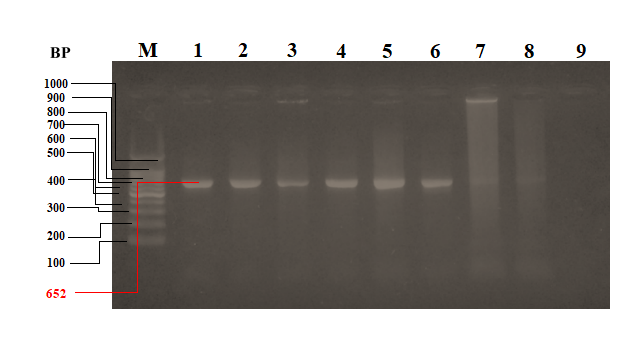


**Supplementary figure 01: Agarose gel electrophoresis of colony PCR products of *E.coli*-BL21-pJET1.2-*CadA/CadR* cells (Modified and cropped image)** Well Numbers: M- 100bp DNA ladder, 2- positive control DNA construct, 3-9 - isolated colonies of *E.coli*-BL21-pJET1.2-*CadA/CadR* cells.


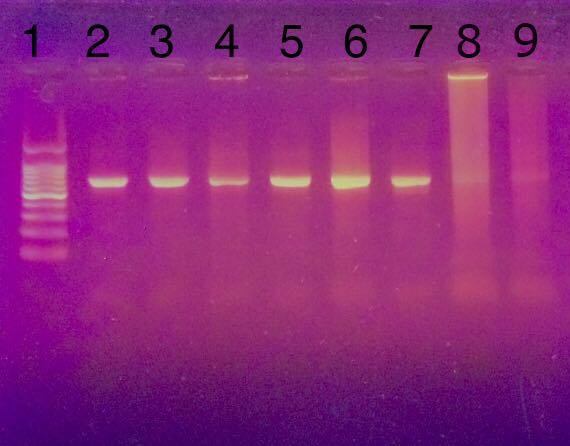


**Supplementary figure 02: An uncropped** **image of agarose gel electrophoresis of colony PCR products of *E. coli*-BL21-pJET1.2-*CadA/CadR* cells** Well Numbers: 1- 100bp DNA ladder, 2- positive control DNA construct, 3-9 - isolated colonies of *E.coli*-BL21-pJET1.2-*CadA/CadR* cells.
